# The Perception of Physician Empathy by Patients with Inflammatory Bowel Disease

**DOI:** 10.1371/journal.pone.0167113

**Published:** 2016-11-22

**Authors:** Costanza Chiapponi, Maxie Witt, Gabriele E. Dlugosch, Veit Gülberg, Matthias Siebeck

**Affiliations:** 1 Department of General, Visceral, Vascular and Transplantation Surgery, Hospital of the University of Munich (LMU), Munich, Germany; 2 Center of Empirical Educational Research (zepf), University of Koblenz-Landau, Campus Landau, Landau in der Pfalz, Germany; 3 Medizinische Klinik IV, Division of Gastroenterology, Hospital of the University of Munich (LMU), Munich, Germany; University Hospital Llandough, UNITED KINGDOM

## Abstract

**Background and Aims:**

This study focused on the difference between perceived and desired physician empathy (pPE and dPE) in the eye of patients with inflammatory bowel disease (IBD). It was investigated if a discrepancy (ΔPE) correlates with trust and satisfaction of patients. At the same time the aim was to gain detailed information about the subjective burden of disease and the resources of IBD patients, in order to better understand them.

**Methods:**

A modified version of the German Version of the Consultation and Relational Empathy (CARE) measure was completed as a paper-and-pencil questionnaire by IBD patients attending our facility (n = 32) and as an online survey by IBD patients at other locations throughout Germany (n = 89). Patients were in average 36.3±12 years old.

**Results:**

The mean (SD) rating of pPE was 3.93 (0.96) on a scale of 1 to 5 (“poor” to “excellent”); however, the mean (SD) dPE was 4.38 (0.48) on the same scale. ΔPE correlated with perceived empathy and with patients’ satisfaction with treatment and trust in their health care providers. Patients reported quite a high subjective burden (mean [SD]: 2.93 [.63]) and named family, friends, and support groups as resources.

**Conclusions:**

Rather than assessing patient satisfaction with treatment and trust in their physician only with perceived PE, we suggest ΔPE as a useful additional parameter.

## Introduction

Inflammatory bowel disease (IBD) represents a group of chronic conditions with a relapsing-remitting course. Most patients are young: 20% of patients develop the first symptoms during childhood, and 5% are diagnosed before their 10th year. In the last century, a psychosomatic origin of the disease was suggested. Nowadays, the pathogenesis is better understood, and a current concept of the disease is a breakdown of the intestinal epithelial barrier with infiltration by cells of the innate and adaptive immune systems, which release a number of cytokines. These cytokines may pass the blood-brain barrier and cause neurologic and behavioral changes [1]. The early onset and need for lifelong treatment are responsible for a high burden of disease and reduced quality of life [2]. Patients often tend to deny their condition and become inconsistent with their schedule of follow-up visits and laboratory tests and less adherent to medical therapy [3]. Consequently, IBD patients represent a challenging group for the treating gastroenterologists and surgeons.

Empathy has received much attention as a research topic in the past decades and yet there is no general agreement about its definition. For this study, we used the concept of empathy defined by Mercer and Reynolds [4]. These authors have identified four components of empathy: an emotional component (the ability to share patients’ feelings), a moral component (the physician’s intrinsic motivation for empathic behavior), a cognitive component (the ability to identify and understand patients’ feelings), and a behavioral component (the physician’s ability to show understanding and even partly share patients’ feelings and the will to find solutions). The fourth component is probably the most important, because it represents the “feedback” that patients actually receive.

There are reports that pain and fear can be reduced if patients perceive high physician empathy (PE) [5]. Also, PE enables patients to better cope with their disease and situation [6]. In cancer patients, high perceived PE correlates with a lower rate of depression and higher satisfaction with treatment [7]. So far, the relevance of PE has been investigated in several studies that focused mainly on cancer patients [8] and diabetes patients [9]. In the recent literature, some studies examined the role of PE in hand and trauma surgery [10, 11]. To our knowledge, however, no study has focused on PE in IBD patients. A PubMed search with the terms “inflammatory bowel disease AND “physician empathy” delivered two results [3, 12]; and a search with the terms “inflammatory bowel disease” AND “empathy,” five [3, 12, 13, 14, 15]. The publication by Burish (2014) reports on the recent ECCO-Epicom study on quality of care in Eastern and Western Europe; one of the items on the questionnaire used in this study was “empathy,” which the study defined as “interest in how IBD impacts the quality of life of patients” and “showing appropriate courtesy” [13].

Most studies have used physician self-reports to measure empathy, but it is not clear how closely such instruments correlate with patients’ perceptions. The Consultation and Relational Empathy (CARE) measure is the only one that assesses empathy from the patients’ perspective [16] and has proven reliability and validity. In this study, we wanted to explore how IBD patients perceive PE (perceived PE, pPE) and how relevant empathy is to them (desired PE, dPE), because PE can be a resource for patients and can even improve their compliance [17]. Consequently, we decided to use a modified, German-language version of the CARE measure [18, 19]. Because we were interested in evaluating whether PE is meeting the expectations of IBD patients, we reworded the items of the CARE measure to gather information about dPE. We assumed that congruence (i.e. alignment or a positive difference) between pPE and dPE would correlate with satisfaction and trust, whereas a negative difference would imply the opposite. Of course, the precondition is that PE is relevant to patients. If PE is low but unimportant to patients, one might find that they are nonetheless satisfied.

Thus, this study aimed to gather more detailed information about IBD patients’ psychosocial stress and resources and their trust in their physicians and satisfaction with their treatment, in order to better understand them and possibly improve the physician-patient partnership (PPP). In addition, we assessed what qualities other than empathy are important to patients, how satisfied they are with their current situation, what subjective burden they carry, and which resources are available to them.

## Methods

A total of 121 participants were included. The sample consisted of two groups of patients: 1) IBD patients presenting as outpatients at our IBD clinic (Hospital of the University of Munich at LMU) were consecutively enrolled in the study from September 2013 to February 2014, n = 32 (26.4%); 2) IBD patients were recruited nationwide from November 2013 to February 2014 through patient support groups identified via the German IBD Network (http://www.kompetenznetz-ced.de), n = 89 (73.5%). A diagnosis of IBD was confirmed in 113 participants (20.4% ulcerative colitis, 76.1% Crohn’s disease); although 3.5% of participants did not yet have a final clear diagnosis, they were included in the analyses because IBD was highly suspected. Thirty patients were male (24.8%) and 91 female (75.2%). Their age ranged from 17 to 80 years (M [SD] = 36.32 [12.74]); 88.4% of participants were German, 56.2% were in a stable relationship, and 62% had no children (Table 1).

**Table 1:** Demographics of the survey population.

		n	%
**gender**	female	91	75%
	male	30	25%
**age**	17–20	8	7%
	21–30	40	33%
	31–40	29	24%
	41–50	23	19%
	51–60	14	12%
	>60 years	5	4%
	no information	2	1%
**nationality**	German	107	88%
	Other	14	12%
**marital status**	Stable partnership	68	56%
	Single	53	44%
**children**	yes	45	62%
	no	75	37%
	no information	1	1%

At the end of their consultation, outpatients at the IBD clinic completed a paper-and-pencil version of the CARE measure anonymously and in a separate room. They also filled out a questionnaire about their demographic information, disease history, previous medical experience, and the characteristics of the consultation they had just received. Specifically, the questionnaire assessed the patients’ overall satisfaction with the visit, the physician’s communication skills, and the degree of empathy shown by their physician. Moreover, patients were asked about the attributes, behaviors, and skills they wish to find in their health care providers, their level of subjective burden and resources, and their previous experiences with other physicians (S1 Questionnaire used for the survery translated into English).

The patients recruited at other locations in Germany completed an online version of the same instruments. Administrators of self-help groups were identified via the German IBD Network and asked to send a link to the instruments to patients on their address lists. Participation was voluntary. Patients were invited to leave their email address if they wished to receive the results of the study. All participants were blinded to any specific study hypothesis, and none of the variables was mentioned in the survey cover letter. They were asked to rate their last consultation with their treating physician.

The CARE measure was first described by Mercer in 2004 [4], then modified and translated into German by Neumann [18, 19]; this study used the latter version. The German CARE measure includes five cognitive and five affective items, which are rated on a Likert scale ranging from 1 to 5 (1 = poor; 5 = excellent). To gather information for our research question, we modified the German version of CARE by introducing two self-developed items that cover particular aspects of IBD and used two different versions of each item to determine pPE and dPE. The two additional items for IBD were: “Is your physician able to let you comfortably speak about embarrassing symptoms of your disease?” and “Does your physician examine you with care and respect?” These two elements were developed on the basis of a literature review, previous qualitative data obtained by the researchers, and advice from a panel of stakeholders.

Additionally, the questionnaire included two open questions that asked patients to describe a relevant negative experience and mention important attributes of a good doctor. Also, it included two items about satisfaction with the physician and three about trust in their physician. These items were also specifically created by the authors of this survey and had not previously been validated; we found a Cronbach’s alpha of .78 for “satisfaction” and .76 for “trust.”

We added an additional seven self-developed items to define a subscale for the subjective burden of IBD. Participants rated every item on a scale of 1 to 4 (1 = “not stressful at all,” 4 = “very stressful”).

In a further, free-text part of the questionnaire, patients were asked to name stressors associated with the disease and resources that helped them cope with it (family and friends, a good relationship with their physician, plans and goals, distraction through sports/activities, relaxation, religion/spirituality, looking for and obtaining information about the disease).

The study was approved by the IRB of the medical faculty of the LMU Munich (Nr. 343–13). Patients gave written informed consent to participate in the study. All authors had access to the study data and reviewed and approved the final manuscript.

### Statistics

Groups and scales were described by means (M) and standard deviations (SD) and by absolute and relative frequencies. Group results were compared with Welch’s or the Mann-Whitney U test for independent samples where appropriate. Normal distribution was tested with the Kolmogorov-Smirnov or Shapiro-Wilk test. Spearman’s rank correlation (rho) was used for correlations. According to Cohen’s classification, r > .10 is a small, r >.30 a medium, and r > .50 a large effect size (S1 Raw Data).

## Results

### Difference (ΔPE) between perceived physician empathy (pPE) and desired physician empathy (dPE)

**Fig 1:**
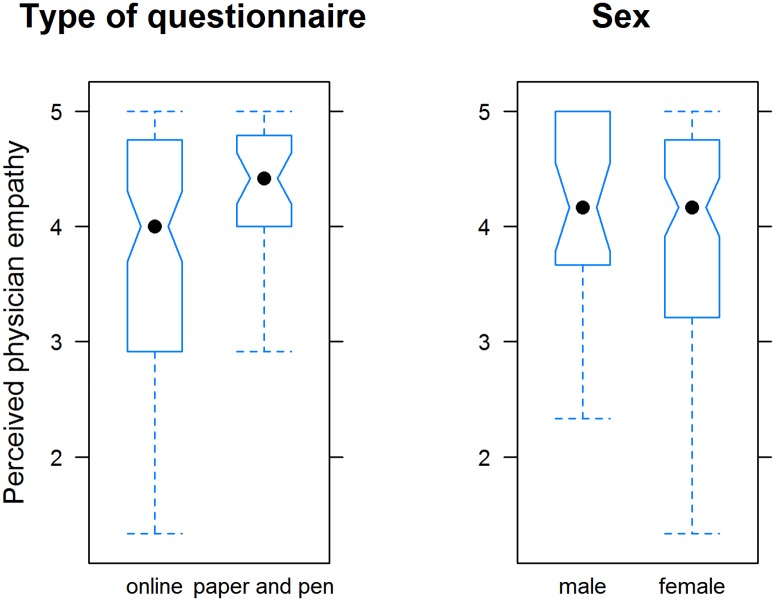
Perceived physician empathy as assessed by the Consultation and Relational Empathy (CARE) measure; Tukey’s box plots. Left: The questionnaire was administered as either an online or a paper-and-pen version; the difference was statistically significant (p <0.001; Mann-Whitney U test). Right: Sex of patients; no difference.

**Fig 2:**
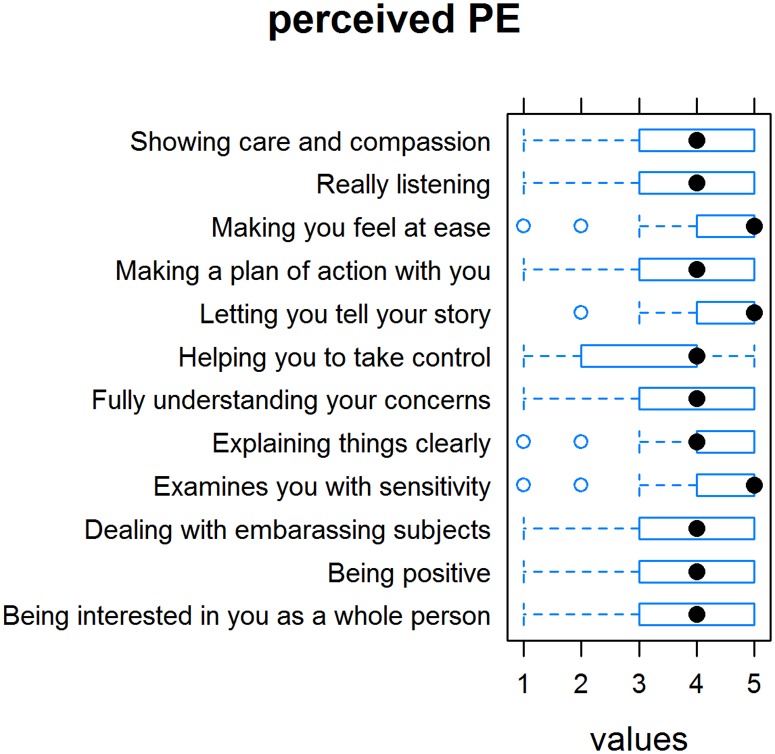
Ratings of “perceived empathy” items on the Consultation and Relational Empathy (CARE) measure.

The mean (SD) pPE score was 3.93 (0.96) (Fig 1). Patients who completed the paper-and-pencil questionnaire rated pPE higher (4.33) than patients who took the online survey (3.80; p<0.001). Men generally rated pPE slightly higher than women (4.15 versus 3.86); however, the difference was not significant. In our modified version of the CARE measure, the scores were highest for the items “Does your physician behave in a way that you feel comfortable with?” and “Does your physician examine you with care and respect?” and lowest for the items “Does your physician help you in finding a way to deal with your disease?” and “Does your physician show interest for you as a person and for your background?” (Fig 2).

**Fig 3:**
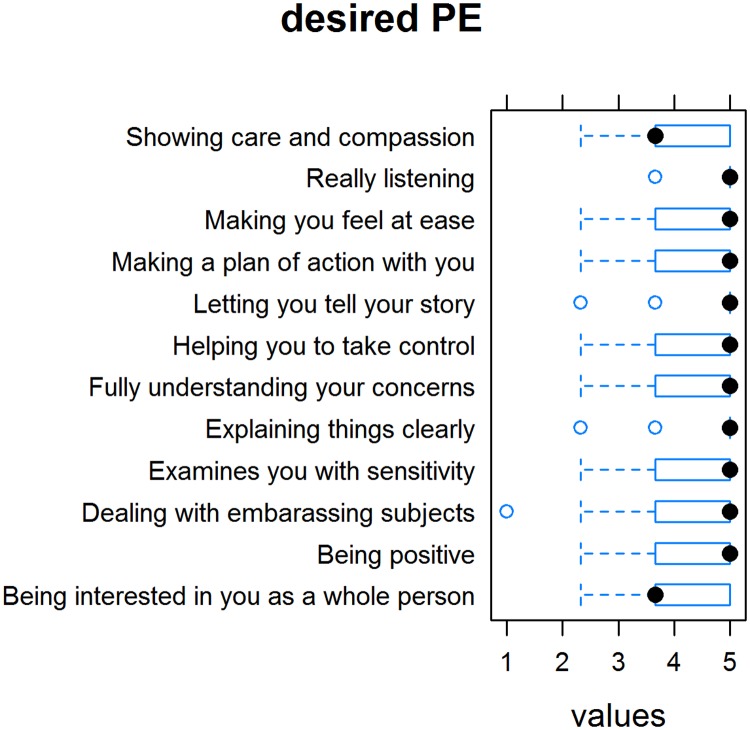
Rating of “desired empathy” items on the Consultation and Relational Empathy (CARE) measure.

The mean (SD) score for dPE was 4.38 (0.48) (Fig 3). We found no differences between the paper-and-pencil and online groups or between men and women. Among the dPE indicators, patients gave the highest rating to the item “The physician really listens to me.” It was significantly more important for women than for men that a physician made them feel comfortable when dealing with embarrassing subjects/examinations.

**Fig 4:**
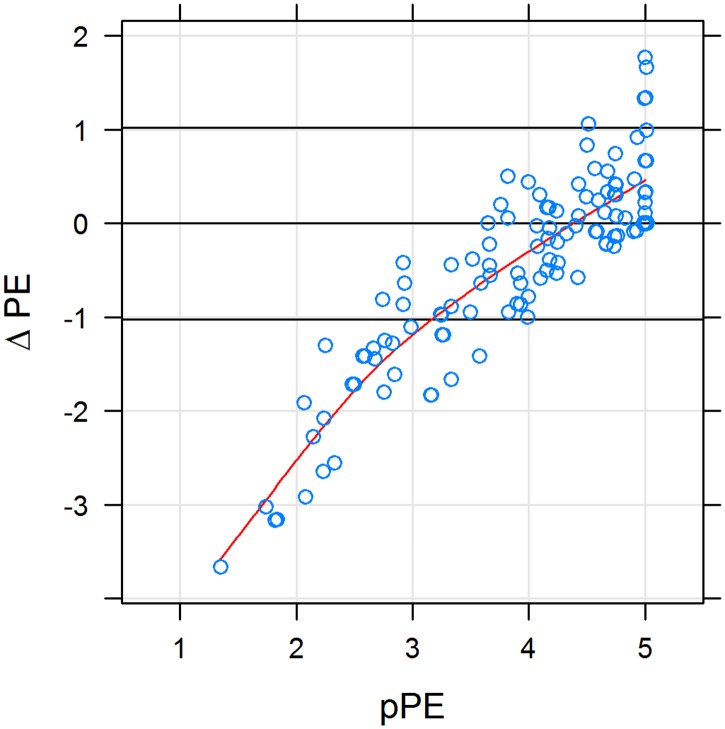
Difference between desired and perceived physician empathy (ΔPE) as a function of perceived physician empathy (pPE). X-Y scatterplot; horizontal lines denote equality between desired and perceived physician empathy plus or minus 1 standard deviation. Red curve is a cubic spline fit. Perceived physician empathy and **Δ**PE were strongly correlated (Spearman’s rho = 0.83; p < 10^−15^). Desired and perceived physician empathy were balanced if perceived physician empathy was > 4. In 28 subjects, **Δ**PE was 1 SD below zero, and in 5 cases it was 1 SD above.

In 28 participants, ΔPE (= pPE-dPE) was 1 SD below zero, meaning that these patients wished to be treated more empathically by their physician than they currently perceived (dPE > pPE; ΔPE < 0; negative difference) (Fig 4). As Fig 1 shows, the higher the pPE score, the smaller the difference between dPE and pPE (ΔPE ≥ 0). In 5 cases, ΔPE was 1 SD above zero, meaning that these patients perceived PE to be higher than they had desired. It goes without saying that patients evaluated such situations positively.

A total of 51 patients (66.2%) reported negative experiences with other physicians in the past. Only 11 (14.3%) reported having only positive experiences. Twelve patients had never seen other health care providers before (15.6%). Twenty patients reported that they had not found previous health care providers to be competent enough, and 14 reported that previous providers had not taken enough time for them. Seven patients felt that they had not been well enough informed about their disease, and four had the impression that the doctor did not listen when they spoke. Table 2 summarizes these answers.

**Table 2:** Free-text responses to questions about patients’ perceptions of previous negative experiences with physicians (translated into English by the authors).

Category	Example responses	*n*
Poor medication/ treatment	“Expensive medications as long-term treatment …Not usually interested in the side effects” “Was always treated for a flu-like infection—for 1½ years” “Not up-to-date with the latest medical information” “They didn’t know how CIBD [*chronic inflammatory bowel disease*] should be treated…sent time and again from one physician to the next”	20
Lack of time	“He never had time (2 minutes at the most)…was always offhand” “Never took time for me…always in a rush and hardly gave me a chance to speak” “It’s not possible for a conversation to develop”	14
Lack of interest; not taken seriously	“Aren’t interested in the patients‘ suffering” “Didn’t take me seriously as a patient … my personal, familial environment was ignored” “Portrayed me as a hypochondriac”	11
Too little information	“Told me I have CD [Crohn’s disease] and then sent me home without giving me any more information” “Physicians don’t give you information about the disease” “Information about Crohn’s disease was poor or sometimes even wrong”	7
Not very empathic	“Very unfriendly and not at all empathic” “Wasn’t considerate during examinations, for example; worries, feelings of embarrassment etc. were ignored” “Weren’t empathic and got examinations over with as quickly as possible”	6
Does not listen	“Didn’t really listen”	4
Change of physician	“CIBD clinic…have to tell everything from the beginning every time” “As soon as I’d got used to a physician … I’m asked to look for another one” “Substitute physicians in a CIBD clinic … don’t know patient and course of illness”	3
Waiting times	“No timely appointments” “Several patients for one appointment time, have to wait for hours”	2
Comprehensibility	“Couldn’t explain anything … threw Latin medical terms around”	1
Miscellaneous	“Past: often very negative” “It was like being in Kindergarten” “Completely dissatisfied” “In 60 years one has all kinds of experiences”	1

### Further attributes and behaviors of a good care provider

Patients were asked to state in free text what is particularly important to them besides empathy (see Table 3 for example responses and the number of responses).

**Table 3:** Patients’ free-text responses to questions about the desired qualities of health care providers (translated into English by the authors).

Category	Example responses	*n*
Accessibility	“In urgent cases also be accessible at short notice” “That you can reach him easily on the phone” “Contact my physician by email to ask important questions”	9
Cooperation/ responsibility of physicians	“Have a maximum of 2 physicians as my main contacts and not…be taken care of by a whole group” “Coordinate care for an individual patient with other physicians, jointly discuss the further approach to treatment” “Better collaboration with other treating physicians (family physician, other specialists)”	9
Individual care/ treatment concept	“Analysis of individual solutions that were developed by patients” “Don’t always recommend surgery straight away, just because it brings money” “Physicians need more freedom to treat patients in the way that’s best for them and not in the way that the health insurance companies determine” “Listen to the patients‘ suggestions … search together for solutions”	8
Shorter wait times, more time for patients	“I wish there was more time for each patient.” “That you can get an appointment sooner when you’re not feeling well, and you don‘t have to wait for hours.”	7
Taking patients seriously	“The feeling of being understood and taken seriously” “The physician should also respect me and not treat me like a hypochondriac”	3
Considers other illnesses	“That he also considers my other illnesses” “See the whole issue of CIBD [*chronic inflammatory bowel disease*] and not just limited to the intestines”	2
Work atmosphere	“Good working atmosphere in the practice” “That he wasn’t dependent on moods”	2
New/alternative treatment methods	“Knowledge of new treatment methods” “Not only be interested in conventional medicine but also in things like acupuncture, kinesiology, etc.”	2
Acceptance	“Physician that gives me the feeling I’m not a hopeless case …” “That my physicians doesn’t treat me like a ‘hopeless case’ but rather as a ‘normal’ patient”	2
No wishes	“None, everything very good.“” “I’m very satisfied.”	2
Miscellaneous	“Parking spaces nearby” “More privacy in hospital”	9

The most common response was wanting their physicians to be more accessible via telephone or email (n = 9) and to cooperate better e.g. with their family practitioner (n = 9). Also, they wanted more time with their provider (n = 7), to have the feeling that they were being taken seriously (n = 3), to receive information from their health care providers about other therapeutic options (n = 2), and for their providers to be open to trying alternative treatments (n = 2).

### Trust in health care providers and satisfaction with treatment

The congruence between pPE and dPE correlated positively with trust and satisfaction (Spearman’s rho = .47 and .32, respectively). Trust and satisfaction were quantified with two and three additional items, respectively.

**Fig 5:**
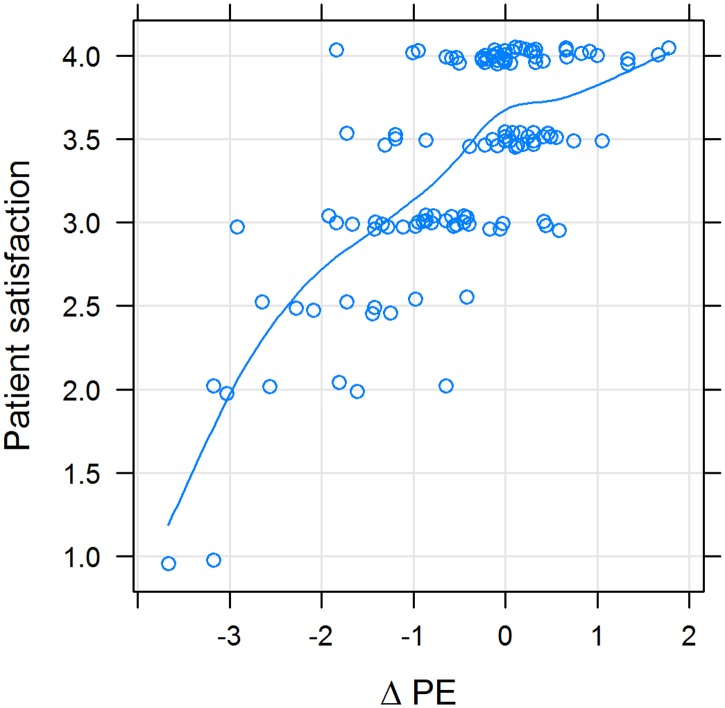
Patient satisfaction with the physician as a function of the difference between desired and perceived physician empathy (ΔPE); X-Y scatterplot. Variables were positively correlated (rho = 0.47; p < 10^−10^). The line represents a cubic smoothing spline fit. If **Δ**PE was negative, patient satisfaction was low and rose steeply; and if it was positive, patient satisfaction rose less steeply.

**Fig 6:**
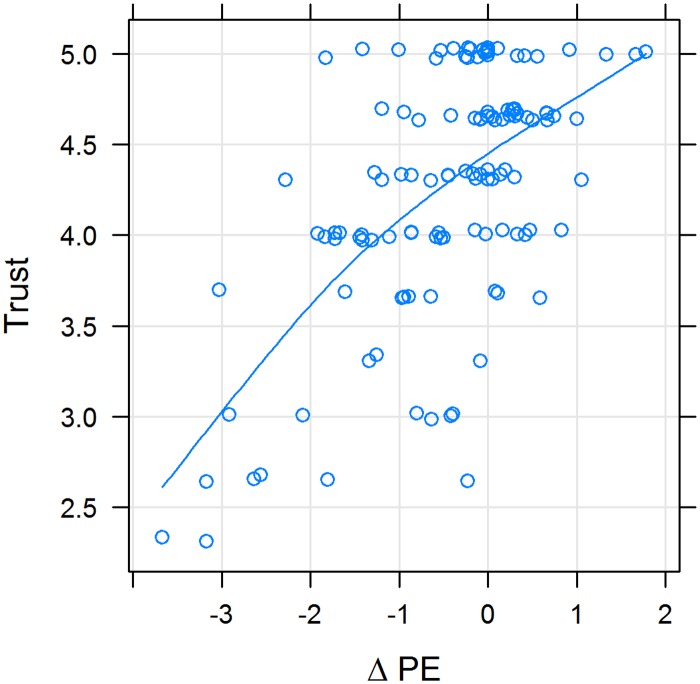
Association between trust in the physician (Trust) and the difference between desired and perceived physician empathy (ΔPE); X-Y scatterplot. Variables were positively correlated (rho = 0.32; p < 10^−7^). The line represents a cubic smoothing spline fit.

**Fig 7:**
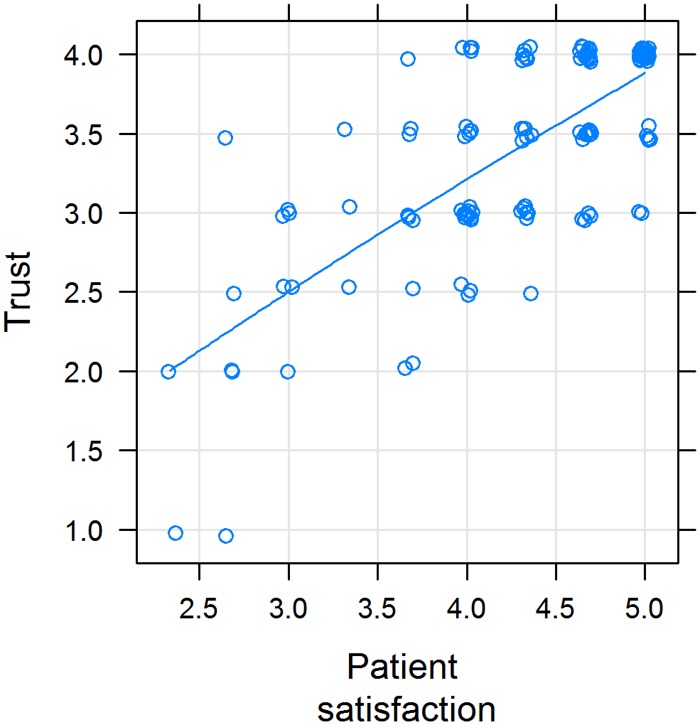
Association between trust in the physician (Trust) and patient satisfaction; X-Y scatterplot. Variables were positively correlated (rho = 0.66; *P* value < 2.2e-^16^). The line represents a cubic smoothing spline fit.

Figs 5 and 6 show that the smaller the difference between pPE and dPE, the higher the rating of satisfaction and trust. Values of ΔPE above zero have less influence on satisfaction and trust, i.e. the slope of the line is less steep (Figs 5 and 6). Also, satisfaction and trust correlate with another (Fig 7).

### Subjective burden and resources of IBD patients

**Fig 8:**
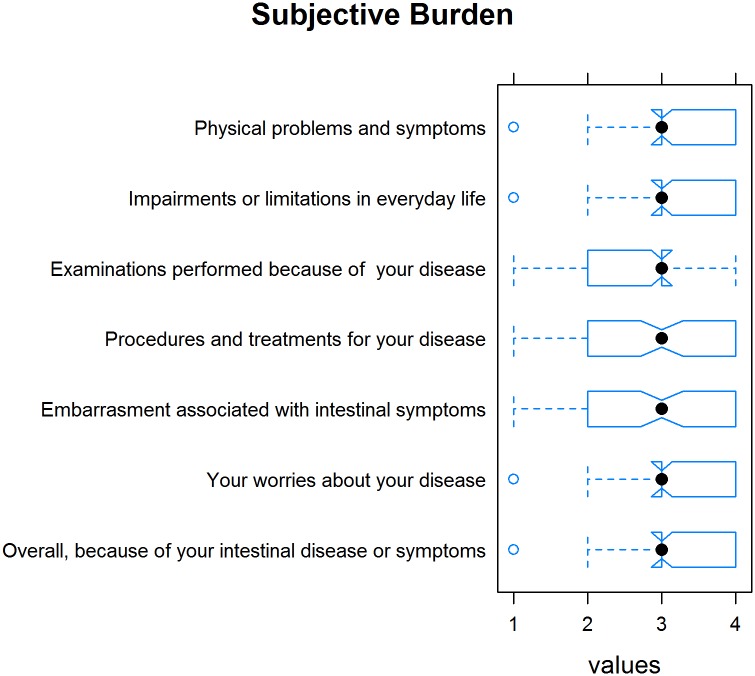
Tukey’s box plots with a notch depicting the subjective burden of various aspects of the disease and their ratings.

The mean (SD) sum of all subjective burden-related items (on a scale of 1 = “not stressed” to 4 = “very stressed”) was 2.93 (.63), indicating that IBD is perceived as quite stressful by patients. Fig 8 depicts the burden-related items and their ratings (Fig 8).

**Fig 9:**
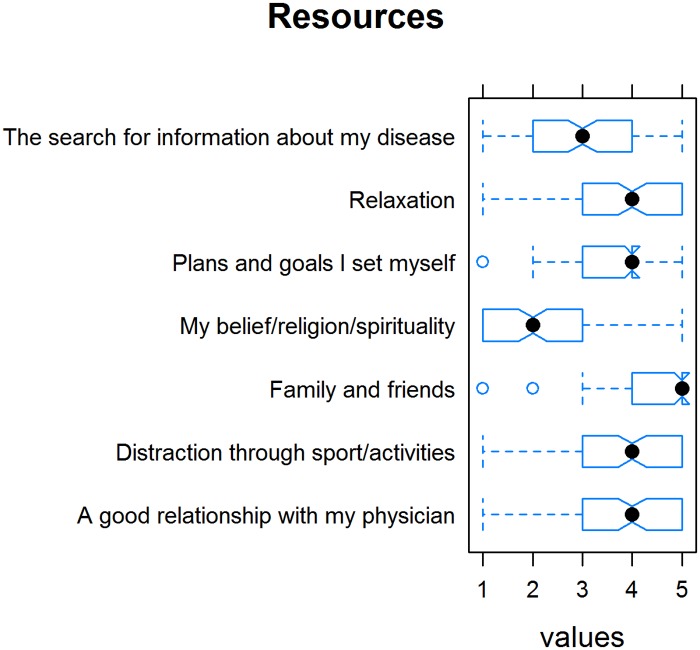
Tukey’s box plots with a notch depicting patient resources and their ratings.

Concerning their resources, 62.8% of patients see “family and friends” as a resource; 36.4%, “a good patient-physician partnership”; and 52.9%, “belief/religion/spirituality.” Ten patients mentioned other patient self-help groups and social media groups as a good resource, seven their hobbies and their pets. Fig 9 summarizes the mean ratings for individual resources. Participants were asked to mention in a free-text item the resources that are particularly important to them. Table 4 summarizes their answers.

**Table 4:** Patients’ free-text responses to questions about their resources for coping with the disease (translated into English by the authors).

Category	Example responses	*n*
Exchange with other people affected	“A Facebook group where someone is always willing to listen” “Forums, Facebook groups, other affected people” “Self-help group”	9
Hobbies	“Pets” “My dog…I walk him for hours” “Creative design”	9
Acceptance	“Don’t work against the disease…accept that it’s there”	8
Distraction	“As far as possible, try not to take the disease too seriously… distract yourself…enjoy life now” “Don’t concern yourself with it too much” “Distraction through my profession”	7
Integration of the disease in everyday life	“Integrate into everyday life” “Good, well-tolerated nutrition” “Integration in day-to-day life”	3
Professional support	“Psychological support, alternative medicine” “Rehabilitation”	2
Positive attitude	“I’m in charge of my life and the disease isn’t” “View the situation positively & don’t let it bring you down”	2
Information search	“Studies and research give hope that there’ll be new knowledge/treatment methods.” “The knowledge how I got the disease and how I can deal with it.”	2
Miscellaneous	“Things that make me happy” “Trust in my physician”	2

## Discussion

To our knowledge, the present study is the first to focus on the role of perceived and desired physician empathy (pPE and dPE, respectively) from the perspective of patients with IBD and on the correlations of PE with trust and satisfaction in this group. We performed a literature research before designing our study and found no similar studies in this field. The aim of our study was to identify both potential ways to optimize the physician-patient partnership (PPP) and a possible incongruence between the current and desired PE in the eye of IBD patients. We chose to concentrate on PE as an important aspect of physician-patient communication and used a modified version of the CARE measure that allowed us to focus more on the patients’ perspective rather than that of physicians.

When asked about the desirable qualities of a good physician, both physicians and patients mentioned empathy [4]. The patients in our study found that their current health care providers are empathic (3.9 of 5); however, they wish that their physicians showed more empathy (4.4 on a scale of 1 to 5). To make sure that this finding is not due to a methodological bias, we asked participants whether they had had any different experiences in the past. Most reported that they had been treated by several different physicians and that they were not satisfied with those experiences. Therefore, we conclude that the high level of PE reported in this survey cannot be extended to all physicians. The results of the ECCO-Epicom study indicate a difference between Eastern and Western Europe [13], but the current study was performed solely in Germany. Moreover the online survey found lower PE values than the paper-and-pencil survey. This might be due to the different time frame in which patients completed the survey (the paper-and-pencil group completed it directly after a consultation; the online group, at some unspecific time) and to the fact that online surveys might appear more “anonymous” than a survey performed at a physician’s office directly after a consultation. However, it is also possible that the physicians involved in this study acted particularly empathically, perhaps because they were aware of the survey. The German version of the CARE measure was validated partly by asking several patients to rate the same physician, whereas in our paper-and-pencil survey patients were asked to rate only the one experience they had just had (instead of rating their health care providers more generally). Several of the on-site patients (n = 32) probably rated the same physician, because only three physicians were involved in this study. It is not possible to identify the physicians who were assessed by the online participants. This difference between the groups might be a limitation of the study.

PE is very relevant to patients. We found a significant positive correlation between the participants’ level of subjective burden and their rating of the relevance of PE, which underlines the role of PE as a resource for patients. Women in particular attached great importance to not feeling embarrassed when they have to talk about classical IBD symptoms such as diarrhea, nausea, abdominal pain, and gas bloating. In this study the physicians being judged in the paper-and-pencil arm were male. The patients in the internet group were not asked to mention the gender of their health care providers. There are commonly no IBD-Nurses in Germany, who might take over these special subjects. Consequently, physicians might learn from this study that it is particularly important to try to reduce female patients’ feelings of embarrassment when they talk about such symptoms. Physicians can encourage patients to speak openly about these subjects by asking appropriate questions, for example. Adequate communication training in medical education could be very helpful.

Competence, information about alternative treatments, and patient-centered treatment were also mentioned as being very important to patients. These findings are similar to those of a recent study on prostate cancer patients undergoing radiation [20]. Additionally, participants mentioned the importance of organizational details such as being able to reach the doctor easily, good communication between their IBD specialist and family practitioner, and waiting times that are not too long. Some of these points were given as an explanation why they had chosen previously to change their treating physician.

We expected to find that patients would be unhappy with their treatment if pPE and dPE were incongruent. Two thirds of patients showed such an incongruence; however, the incongruence seems only to be relevant when dPE is higher than pPE (i.e. when ΔPE is negative). This was the case in 27.3% of patients. In all other cases, pPE was the same or even higher than the dPE. The congruence between pPE and dPE correlated positively with trust and satisfaction. Trust and satisfaction also correlated positively with each other. These findings are similar to those of a recent Norwegian study on quality of care in IBD patients, which found a high satisfaction rate (86%) [21]. Dissatisfaction was mostly related to communication aspects, showing the need for improvement of physician-patient communication.

In our study, patients identified IBD as a source of subjective burden: the mean subjective burden was 2.9 on a scale of 1 to 4. IBD patients often have depression [22]. Interestingly, a recent study reported that depression has a similar pathophysiology to that of IBD, i.e. they are both clinical expressions of activated immune-inflammatory, oxidative, and nitric oxid stress pathways, which may explain the frequent co-occurrence of these two conditions [23]. Another recent study found a strong relationship between perceived stress and gastrointestinal symptoms [24]. This association might explain our finding that the level of subjective burden correlated negatively with patient satisfaction and positively with the importance of PE for patients. “A good patient-physician partnership” (PPP) was a resource in just over a third of the IBD patients in the present study. Therefore, strategies aimed at improving the PPP might help patients to better bear the burden of disease and might increase their satisfaction with their treatment and PPP. PE seems to be able to influence pain, fear [25], how well patients cope with their disease [26], and cancer patients’ levels of depression and satisfaction with life [18]. For this reason, we focused on gathering information about how patients perceive their current PPP, particularly the empathy of their health care providers.

To the best of our knowledge, this study is to the first to evaluate ΔPE in IBD patients. A strength of the study is the use of a patient-centered measure, the CARE. The study does have various limitations, though. First, the generalization of our findings to other hospitals is limited, because we included patients at only one hospital. The additional use of an online survey improves the generalizability. However, the internet may allow patients to give a more liberal evaluation, because they do not have to worry that their physician will learn about their opinions. Therefore, the comparison of paper-and-pencil survey and online results might be biased. The recent study by the ECCO-Epicom group found a significant difference between Eastern and Western Europe, so our result can probably be seen to be relevant only in Germany [13]. Furthermore, generalization within Germany also is limited because of the fairly small sample size. In addition, the study did not take into account confounders of the relationship between physicians and patients such as type of health insurance, income level, common culture of physician and patient, and the patient’s social environment. For example, physicians have been found to spend more time with patients who have private health insurance, which may influence patients’ perception of PE [27]. The CARE is a validated method to measure physician empathy. However, the German version used in this study has been validated only in cancer patients. Furthermore, we added two items to evaluate the particular needs of IBD patients; although these items were not validated, they had a Cronbach’s alpha of .96. Also, the items used to measure satisfaction and trust were formulated for the test and were not previously validated; the Cronbach’s alpha was .76 and .78 for these items, respectively. A further potential bias is that the on-site physicians were aware that the survey was being performed and consequently might have behaved more empathically than usual.

## Conclusion

We identified a wide range of personal attributes, behaviors, and skills of IBD specialist physicians that can make a real difference to patient care. The difference between desired and perceived PE might predict satisfaction with and trust in the treating physician. This aspect needs to be considered in medical education and daily clinical practice.

## Supporting Information

S1 Questionnaire Questionnaire used for the survey translated into English. (DOCX)

S1 Raw Data (DOCX)
