# Supplementary material for: The Perception of Physician Empathy by Patients with Inflammatory Bowel Disease
Source: PLoS One. 2016 Nov 22;11(11):e0167113. doi: 10.1371/journal.pone.0167113 (PMC5119824; doi:10.1371/journal.pone.0167113)
Supplement: S1 Questionnaire — (DOCX) [file pone.0167113.s001.docx]

1. **Please evaluate your current physician by responding to the following questions.**

Please choose only *one* response for *each* question!

|  | Always | Mostly | Sometimes | Hardly ever | Never |
| --- | --- | --- | --- | --- | --- |
| **Does the physician behave in a way that lets you feel comfortable in his presence?**  (He is friendly, caring and respectful, not distant or curt.) | ❑ | ❑ | ❑ | ❑ | ❑ |
| **Does the physician take time for you?**  (He gives you time to describe your disease in detail. He doesn’t interrupt or distract you.) | ❑ | ❑ | ❑ | ❑ | ❑ |
| **Does the physician really listen to you?**  (He pays full attention to what you say and isn’t distracted while you talk.) | ❑ | ❑ | ❑ | ❑ | ❑ |
| **Is the physician interested in you as a person and in your environment?**  (He knows about important details of your life or your personal situation or he asks about them and he doesn’t treat you like a “number.”) | ❑ | ❑ | ❑ | ❑ | ❑ |
| **Does the physician really understand your concerns?**  (He gives you the feeling that he really understands your worries. He doesn’t overlook or gloss over anything.) | ❑ | ❑ | ❑ | ❑ | ❑ |
| **Is the physician empathetic and does he show compassion?** (He really takes care of you and behaves like a person towards you and is not indifferent or distanced.) | ❑ | ❑ | ❑ | ❑ | ❑ |
| **Does the physician encourage you?**  (He has an optimistic attitude. He is honest, but doesn’t have a negative attitude towards your problems. He tries to encourage you rather than lecture you.) | ❑ | ❑ | ❑ | ❑ | ❑ |
| **Does the physician explain everything to you clearly?**  (He fully answers your questions and explains everything clearly. He gives you enough information and doesn’t leave you in the dark.) | ❑ | ❑ | ❑ | ❑ | ❑ |
| **Does the physician help you find a way to deal with your disease?**  (He helps you explore what you can do to improve your health status.) | ❑ | ❑ | ❑ | ❑ | ❑ |
| **Does the physician draw up a treatment plan together with you?**  (He discusses treatment options with you and involves you in decisions – if you want to be involved. He doesn’t ignore your point of view.) | ❑ | ❑ | ❑ | ❑ | ❑ |
| **Does the physician manage to take away the embarrassment of talking about particular symptoms of your disease?**  (He gives you the feeling that you can talk openly to him about your symptoms and that it’s not awkward.) | ❑ | ❑ | ❑ | ❑ | ❑ |
| **During examinations, does the physician deal with you in sensitively and considerately?**  (During examinations, he is careful and empathetic.) | ❑ | ❑ | ❑ | ❑ | ❑ |

1. **Regarding these aspects, what have been your experiences with other physicians in the past? Are there differences to the physician currently treating you? If so, what are the differences?**

Please just write down everything you can think of below!

________________________________________________________________________________________________________________________________________________

1. **Please now respond to the following questions or statements, again with respect to the physician currently treating you.**

Please choose only *one* response for *each* question!

|  | Very happy | Happy | Not so happy | Unhappy |
| --- | --- | --- | --- | --- |
| How happy are you with your physician? | ❑ | ❑ | ❑ | ❑ |
| How happy are you with the treatment you receive? | ❑ | ❑ | ❑ | ❑ |

|  | Strongly agree | Mostly agree | Neither agree nor disagree | Disagree | Strongly disagree |
| --- | --- | --- | --- | --- | --- |
| I trust my physician’s medical opinion. | ❑ | ❑ | ❑ | ❑ | ❑ |
| I always follow my physician’s advice. | ❑ | ❑ | ❑ | ❑ | ❑ |
| I trust my physician overall. | ❑ | ❑ | ❑ | ❑ | ❑ |

1. **Please rate how important the following aspects are to you.**

Please choose only *one* response for *each* statement!

|  | Very important | Important | Less important | Unimportant |
| --- | --- | --- | --- | --- |
| The physician behaves in a way that lets you feel comfortable in his presence. | ❑ | ❑ | ❑ | ❑ |
| The physician takes time for you. | ❑ | ❑ | ❑ | ❑ |
| The physician really listens to you. | ❑ | ❑ | ❑ | ❑ |
| The physician is interested in you as a person and in your environment. | ❑ | ❑ | ❑ | ❑ |
| The physician really understands your concerns. | ❑ | ❑ | ❑ | ❑ |
| The physician is empathetic and shows compassion. | ❑ | ❑ | ❑ | ❑ |
| The physician encourages you. | ❑ | ❑ | ❑ | ❑ |
| The physician explains everything to you clearly. | ❑ | ❑ | ❑ | ❑ |
| The physician helps you to find a way to deal with your disease. | ❑ | ❑ | ❑ | ❑ |
| The physician draws up a treatment plan together with you. | ❑ | ❑ | ❑ | ❑ |
| The physician manages to take away the embarrassment of talking about the particular symptoms of your disease. | ❑ | ❑ | ❑ | ❑ |
| The physician deals with you sensitively and considerately. | ❑ | ❑ | ❑ | ❑ |

1. **We would now like to give you the chance to provide more information about things that haven’t been mentioned yet. What else is important to you with regard to your physician? When dealing with your physician, what would you like (even) more of?**

Please just write down everything you can think of below!

________________________________________________________________________________________________

1. **Please now rate how much of a burden your intestinal disease or symptoms are for you.**

Please choose only *one* response for *each* question!

|  | A heavy burden | A burden | Not much of a burden | No burden |
| --- | --- | --- | --- | --- |
| How much of a burden are your **physical problems** and symptoms? | ❑ | ❑ | ❑ | ❑ |
| How much of a burden are the **impairments** or limitations in everyday life? | ❑ | ❑ | ❑ | ❑ |
| How much of a burden are the **examinations** that are performed because of your disease? | ❑ | ❑ | ❑ | ❑ |
| How much of a burden are the **procedures** and treatments that have to be performed because of your disease? | ❑ | ❑ | ❑ | ❑ |
| How much of a burden is the **embarrassment** associated with the intestinal disease or symptoms? | ❑ | ❑ | ❑ | ❑ |
| How much of a burden are the **worries** that you have about your disease? | ❑ | ❑ | ❑ | ❑ |
| How much of a burden, overall, are the intestinal disease or symptoms in your life? | ❑ | ❑ | ❑ | ❑ |

1. **Now we would like to know what helps you to deal with your disease.**

Please choose only *one* response for *each* statement!

| In dealing with my disease I am helped by …. | Always | Mostly | Sometimes | Hardly ever | Never |
| --- | --- | --- | --- | --- | --- |
| … family and friends. | ❑ | ❑ | ❑ | ❑ | ❑ |
| … a good relationship with my physician. | ❑ | ❑ | ❑ | ❑ | ❑ |
| ... plans and goals I set myself. | ❑ | ❑ | ❑ | ❑ | ❑ |
| … distraction through sport/activities. | ❑ | ❑ | ❑ | ❑ | ❑ |
| … relaxation. | ❑ | ❑ | ❑ | ❑ | ❑ |
| … my belief/religion/spirituality. | ❑ | ❑ | ❑ | ❑ | ❑ |
| ... the search for information about my disease. | ❑ | ❑ | ❑ | ❑ | ❑ |
| … other things, i.e.:  _______________________________________  _______________________________________  _______________________________________ | | | | | |

1. **Finally, we would like to ask you a few questions about you and the history of your disease.**

For *each* question, please choose the appropriate response.

Please note that the following questions do not endanger your anonymity.

1. Are you

❑ male or

❑ female?

1. How old are you? ________ years (Please write your age.)
2. Are you a German citizen?

❑ yes

❑ no, I‘m __________________ (Please write your citizenship.)

1. What is your marital status?

❑ married / long-term relationship

❑ not married / not in a long-term relationship

❑ divorced / separated

❑ widowed

1. Do you have children?

❑ no

❑ yes, I have ____________ (Please write down how many children you have.)

1. What is the highest general education *school qualification* you have achieved?

❑ left school without graduating

❑ basic school qualification (“Hauptschulabschluss”)

❑ intermediate school certificate (“Realschulabschluss,” “Mittlere Reife”)

❑ general higher education entrance qualification (“Fachhochschulreife“, “Fachoberschule,” “Abitur”)

❑ still in school

1. What is the highest *vocational* qualification you have achieved?

❑ no vocational qualification

❑ completed vocational qualification at a company / vocational school

❑ completed education at a technical or vocational school (“Fachschule,” “Meisterschule,” “Technikerschule,” “Berufsakademie,” or “Fachakademie”)

❑ university degree, university of applied sciences degree (z.B. Bachelors, Masters, “Diploma,” “Magister,” State exams)

❑ PhD

❑ still in vocational training (apprentice, intern, student)

❑ other vocational qualification: ­­­­­­­­_________________________ (Please write the name of the qualification here.)

1. Do you already have a confirmed diagnosis for your intestinal disease or problems? If so, please provide the name of the diagnosis.

❑ No, I don’t have a diagnosis yet.

❑ Yes, my diagnosis is:

❑ Ulcerative colitis:

🡪 Do you currently have an acute episode? ❑ Yes ❑ No

❑ Crohn’s disease:

🡪 Do you currently have an acute episode? ❑ Yes ❑ No

❑ Infection, e.g. fistula, abscess

❑ Tumor

❑ Functional disorder

❑ Other: __________________________ (Please write the name of the diagnosis here.)

1. If yes: When were you given the diagnosis?

_______________________ (Please write down the month and year.)

1. How “severe” do you rate your disease on a scale of 1 (mild) to 5 (severe)?

1 2 3 4 5

❑ ❑ ❑ ❑ ❑

mild severe

1. Which and how many of the following examinations have you had so far because of your intestinal problems? (Please check everything that applies and write the number of times.)

❑ Physical examination, number:_______

❑ Rectal palpation, number:_______

❑ Proctoscopy (rectoscopy), number:_______

❑ Sigmoidoscopy (coloscopy of the lower part of the colon), number:_______

❑ Coloscopy (colonoscopy), number:_______

❑ Ultrasound examination of the rectal sphincter, number:_______

❑ Ultrasound examination of the abdomen, number:_______

❑ Blood sampling, number:_______

❑ Computer tomography, number:_______

❑ Magnetic resonance imaging, number:_______

❑ Other X-ray examinations, number:_______

1. Which of the following treatments and how many of them have been performed so far because of your intestinal problems?

❑ Examination under anesthesia, number:_______

❑ Abscess opening, number:_______

❑ Placement of an incision drainage, number:_______

❑ Closure of anal fistula, number:_______

❑ Abdominal surgery for removal of part of the colon, number:_______

❑ Abdominal surgery with attachment of an artificial anus, number:_______

❑ Infusion of medications, number:_______

❑ Widening of a constriction in the anus or rectum, number:_______

1. Have you been diagnosed with other physical or mental diseases?

❑ no

❑ yes, physical diseases:

___________________________________________________________________________

_____________________________________________ (Please write down the diagnoses.)

❑ yes, mental diseases:

___________________________________________________________________________

_____________________________________________ (Please write down the diagnoses.)

**Thank you again for your support!**
