# Supplementary material for: The Perception of Physician Empathy by Patients with Inflammatory Bowel Disease
Source: PLoS One. 2016 Nov 22;11(11):e0167113. doi: 10.1371/journal.pone.0167113 (PMC5119824; doi:10.1371/journal.pone.0167113)
Supplement: S1 Raw Data — (DOCX) [file pone.0167113.s002.docx]

# Raw Data

This file contains the raw data for each of the figures as comma separated values. The first line of each table contains the variable names

## Fig 1: Perceived physician empathy as assessed by the Consultation and Relational Empathy (CARE) measure; Tukey’s box plots. Left: The questionnaire was administered as either an online or a paper-and-pen version. Right: Sex of patients

Raw data from which the visualisation was constructed:

"Case","perceived.PE","Type.of.questionnaire","sex"

"1",3.33333333333333,"paper and pen","female"

"2",5,"paper and pen","male"

"3",4.75,"paper and pen","female"

"4",5,"paper and pen","female"

"5",3.33333333333333,"paper and pen","female"

"6",4.41666666666667,"paper and pen","female"

"7",4.41666666666667,"paper and pen","female"

"8",4.91666666666667,"paper and pen","female"

"9",4,"paper and pen","male"

"10",5,"paper and pen","male"

"11",4.5,"paper and pen","male"

"12",4.16666666666667,"paper and pen","male"

"13",3.91666666666667,"paper and pen","male"

"14",5,"paper and pen","male"

"15",4.75,"paper and pen","male"

"16",4.58333333333333,"paper and pen","female"

"17",4.66666666666667,"paper and pen","female"

"18",4.33333333333333,"paper and pen","female"

"19",4.08333333333333,"paper and pen","male"

"20",4.58333333333333,"paper and pen","female"

"21",5,"paper and pen","male"

"22",4.25,"paper and pen","female"

"23",2.91666666666667,"paper and pen","female"

"24",4.08333333333333,"paper and pen","female"

"25",3.33333333333333,"paper and pen","male"

"26",4.66666666666667,"paper and pen","male"

"27",3.66666666666667,"paper and pen","female"

"28",3.66666666666667,"paper and pen","male"

"29",5,"paper and pen","male"

"30",4.25,"paper and pen","male"

"31",4,"paper and pen","male"

"32",4.83333333333333,"paper and pen","female"

"33",4.75,"online","male"

"34",4.25,"online","female"

"35",3.58333333333333,"online","female"

"36",5,"online","female"

"37",2.66666666666667,"online","female"

"38",4.16666666666667,"online","female"

"39",5,"online","female"

"40",4.66666666666667,"online","female"

"41",5,"online","male"

"42",3.16666666666667,"online","female"

"43",2.08333333333333,"online","female"

"44",3.91666666666667,"online","female"

"45",2.5,"online","female"

"46",4.66666666666667,"online","female"

"47",3.66666666666667,"online","female"

"48",3.16666666666667,"online","female"

"49",4.75,"online","female"

"50",4.75,"online","female"

"51",4.91666666666667,"online","female"

"52",1.83333333333333,"online","female"

"53",4,"online","male"

"54",1.75,"online","female"

"55",2.5,"online","female"

"56",5,"online","female"

"57",4.16666666666667,"online","male"

"58",4.75,"online","female"

"59",3.58333333333333,"online","female"

"60",2.58333333333333,"online","female"

"61",2.91666666666667,"online","male"

"62",5,"online","female"

"63",4.91666666666667,"online","female"

"64",4.41666666666667,"online","female"

"65",5,"online","male"

"66",4.5,"online","female"

"67",3.25,"online","female"

"68",4.41666666666667,"online","female"

"69",4.08333333333333,"online","female"

"70",2.75,"online","female"

"71",3.5,"online","female"

"72",4.58333333333333,"online","female"

"73",5,"online","female"

"74",4.75,"online","female"

"75",3.66666666666667,"online","male"

"76",3.83333333333333,"online","female"

"77",5,"online","female"

"78",1.33333333333333,"online","female"

"79",2.83333333333333,"online","female"

"80",5,"online","female"

"81",2.25,"online","female"

"82",2.75,"online","female"

"83",4.16666666666667,"online","female"

"84",4.25,"online","female"

"85",2.08333333333333,"online","female"

"86",2.83333333333333,"online","male"

"87",3,"online","female"

"88",3.91666666666667,"online","female"

"89",4.16666666666667,"online","male"

"90",4.5,"online","female"

"91",4.66666666666667,"online","female"

"92",5,"online","female"

"93",3.5,"online","female"

"94",3.25,"online","female"

"95",3.75,"online","female"

"96",4.08333333333333,"online","female"

"97",2.91666666666667,"online","male"

"98",4.75,"online","female"

"99",4.91666666666667,"online","female"

"100",2.66666666666667,"online","female"

"101",3.83333333333333,"online","female"

"102",2.25,"online","female"

"103",2.25,"online","female"

"104",2.58333333333333,"online","female"

"105",3.91666666666667,"online","female"

"106",5,"online","female"

"107",5,"online","male"

"108",5,"online","female"

"109",3.25,"online","female"

"110",2.75,"online","male"

"111",4.75,"online","female"

"112",4.75,"online","male"

"113",3.83333333333333,"online","female"

"114",4.58333333333333,"online","female"

"115",5,"online","female"

"116",2.16666666666667,"online","female"

"117",4.75,"online","female"

"118",4.16666666666667,"online","female"

"119",3.25,"online","female"

"120",1.83333333333333,"online","female"

"121",2.33333333333333,"online","male"

## Fig 2: Ratings of “perceived empathy” items on the Consultation and Relational Empathy (CARE) measure

Raw data from which the visualisation was constructed:

"Case","Making.you.feel.at.ease","Letting.you.tell.your.story","Really.listening","Being.interested.in.you.as.a.whole.person","Fully.understanding.your.concerns","Showing.care.and.compassion","Being.positive","Explaining.things.clearly","Helping.you.to.take.control","Making.a.plan.of.action.with.you","Dealing.with.embarassing.subjects","Examines.you.with.sensitivity"

"1",4,4,4,2,3,3,4,3,2,3,4,4

"2",5,5,5,5,5,5,5,5,5,5,5,5

"3",5,5,5,5,4,4,4,5,5,5,5,5

"4",5,5,5,5,5,5,5,5,5,5,5,5

"5",4,3,3,4,3,3,3,4,3,3,3,4

"6",5,5,5,4,4,4,4,5,4,4,4,5

"7",5,5,5,4,4,4,4,5,4,4,4,5

"8",5,5,5,4,5,5,5,5,5,5,5,5

"9",5,5,5,3,3,4,3,4,3,3,5,5

"10",5,5,5,5,5,5,5,5,5,5,5,5

"11",5,5,5,4,4,4,5,5,4,3,5,5

"12",4,5,5,4,3,4,3,5,3,4,5,5

"13",5,4,5,3,4,4,3,4,3,2,5,5

"14",5,5,5,5,5,5,5,5,5,5,5,5

"15",4,5,4,5,5,5,5,4,5,5,5,5

"16",5,5,5,5,4,5,5,4,4,4,4,5

"17",5,5,5,4,5,5,4,4,4,5,5,5

"18",5,5,4,4,3,4,5,4,4,5,4,5

"19",5,4,4,4,4,4,4,4,4,4,4,4

"20",5,5,5,5,4,4,4,5,3,5,5,5

"21",5,5,5,5,5,5,5,5,5,5,5,5

"22",4,5,5,4,4,3,5,4,4,4,4,5

"23",3,2,2,3,1,3,3,4,2,2,5,5

"24",4,5,5,5,4,3,4,5,3,3,4,4

"25",4,4,4,3,4,3,4,4,4,3,4,3

"26",4,5,4,5,5,5,4,5,4,5,5,5

"27",4,4,4,3,3,3,4,4,4,3,4,4

"28",4,5,4,4,4,3,3,3,3,4,4,3

"29",5,5,5,5,5,5,5,5,5,5,5,5

"30",4,5,5,3,4,3,5,5,3,5,5,4

"31",5,5,4,3,4,5,3,4,3,2,5,5

"32",5,5,5,5,4,5,5,5,4,5,5,5

"33",5,5,5,5,4,4,5,5,5,5,4,5

"34",5,4,4,4,5,5,5,3,2,5,4,5

"35",4,4,4,3,4,3,4,4,3,3,3,4

"36",5,5,5,5,5,5,5,5,5,5,5,5

"37",3,3,3,3,2,3,2,2,2,2,3,4

"38",4,4,5,5,4,4,4,5,3,4,3,5

"39",5,5,5,5,5,5,5,5,5,5,5,5

"40",5,4,4,5,5,5,5,5,4,4,5,5

"41",5,5,5,5,5,5,5,5,5,5,5,5

"42",3,4,5,2,2,2,3,3,2,3,4,5

"43",2,2,2,1,2,3,1,2,1,4,2,3

"44",5,4,4,5,3,3,3,4,4,4,4,4

"45",3,3,3,1,2,3,3,2,3,2,3,2

"46",4,5,4,5,5,5,5,4,4,5,5,5

"47",4,4,4,3,3,3,3,5,3,4,4,4

"48",5,4,4,4,4,4,3,4,3,2,3,3

"49",5,5,5,5,5,5,4,4,5,4,5,5

"50",5,5,5,5,4,5,4,5,4,5,5,5

"51",5,5,4,5,5,5,5,5,5,5,5,5

"52",2,3,3,1,1,1,2,2,2,1,2,2

"53",5,5,5,5,4,4,5,5,4,3,4,4

"54",2,2,3,1,1,1,3,1,1,3,2,1

"55",4,4,3,1,1,2,2,4,1,1,2,5

"56",5,5,5,5,5,5,5,5,5,5,5,5

"57",4,5,5,4,4,4,4,5,3,4,5,3

"58",5,5,5,4,5,4,5,5,4,5,5,5

"59",5,5,5,5,4,4,3,4,2,2,2,2

"60",3,2,3,2,2,2,2,3,2,4,3,3

"61",4,4,4,2,2,2,2,4,2,1,4,4

"62",5,5,5,5,5,5,5,5,5,5,5,5

"63",5,5,5,5,5,5,5,5,5,5,4,5

"64",5,5,4,2,4,5,5,5,3,5,5,5

"65",5,5,5,5,5,5,5,5,5,5,5,5

"66",5,4,4,5,4,5,4,5,4,5,5,4

"67",4,4,3,2,3,4,2,4,2,2,4,5

"68",5,5,4,4,4,4,4,4,4,5,5,5

"69",5,5,4,3,3,5,5,5,2,2,5,5

"70",3,2,3,3,2,3,3,2,2,2,4,4

"71",4,4,3,3,4,3,4,4,2,4,3,4

"72",5,5,5,4,4,4,3,5,5,5,5,5

"73",5,5,5,5,5,5,5,5,5,5,5,5

"74",5,5,5,4,5,5,5,5,4,4,5,5

"75",5,4,3,3,4,4,4,5,2,2,4,4

"76",3,4,4,3,4,3,5,5,2,5,4,4

"77",5,5,5,5,5,5,5,5,5,5,5,5

"78",1,3,1,1,1,2,1,1,1,2,1,1

"79",4,3,3,3,3,2,3,2,2,3,2,4

"80",5,5,5,5,5,5,5,5,5,5,5,5

"81",3,3,2,1,3,3,3,3,1,2,1,2

"82",3,3,3,2,2,3,4,3,2,2,3,3

"83",5,4,4,4,5,5,4,4,4,3,4,4

"84",5,5,5,1,4,5,5,5,1,5,5,5

"85",3,3,2,1,2,1,3,2,2,2,1,3

"86",4,3,3,2,3,3,4,2,2,2,2,4

"87",4,4,3,4,3,3,3,3,3,2,1,3

"88",4,5,5,4,4,3,3,3,4,3,5,4

"89",5,5,4,3,4,5,4,4,3,4,4,5

"90",5,5,5,5,4,4,3,4,4,5,5,5

"91",5,5,5,5,4,5,4,4,5,4,5,5

"92",5,5,5,5,5,5,5,5,5,5,5,5

"93",4,3,5,2,3,4,5,4,1,1,5,5

"94",4,4,3,2,2,4,4,4,4,3,3,2

"95",4,4,4,4,4,4,3,3,4,4,3,4

"96",5,4,5,3,4,4,4,5,4,4,3,4

"97",3,2,3,2,3,3,2,4,2,4,4,3

"98",5,5,5,5,5,5,5,5,4,4,5,4

"99",5,5,5,5,5,5,5,5,4,5,5,5

"100",3,3,2,3,2,2,3,3,2,3,2,4

"101",4,4,4,4,4,4,4,3,3,4,4,4

"102",3,2,2,2,2,2,2,2,1,3,3,3

"103",2,2,2,1,1,2,4,4,1,1,3,4

"104",3,4,3,1,2,3,1,5,1,2,1,5

"105",5,4,4,3,4,4,4,4,4,3,4,4

"106",5,5,5,5,5,5,5,5,5,5,5,5

"107",5,5,5,5,5,5,5,5,5,5,5,5

"108",5,5,5,5,5,5,5,5,5,5,5,5

"109",3,4,4,2,2,2,4,5,4,3,2,4

"110",4,3,3,2,2,4,3,4,2,1,1,4

"111",5,5,5,5,4,4,4,5,5,5,5,5

"112",5,5,5,4,5,5,4,5,4,5,5,5

"113",4,4,4,3,4,4,4,5,3,2,4,5

"114",5,5,5,5,4,5,5,3,3,5,5,5

"115",5,5,5,5,5,5,5,5,5,5,5,5

"116",3,4,3,1,1,3,1,1,1,1,4,3

"117",5,5,5,5,5,5,4,5,4,4,5,5

"118",5,4,4,4,4,4,4,4,4,4,4,5

"119",4,4,3,3,4,2,3,4,3,3,4,2

"120",2,2,2,2,2,2,2,2,2,1,2,1

"121",3,2,3,2,3,3,2,2,2,2,2,2

## Fig 3: Rating of “desired empathy” items on the Consultation and Relational Empathy (CARE) measure

Raw data from which the visualisation was constructed:

"Case","Making.you.feel.at.ease.1","Letting.you.tell.your.story.1","Really.listening.1","Being.interested.in.you.as.a.whole.person.1","Fully.understanding.your.concerns.1","Showing.care.and.compassion.1","Being.positive.1","Explaining.things.clearly.1","Helping.you.to.take.control.1","Making.a.plan.of.action.with.you.1","Dealing.with.embarassing.subjects.1","Examines.you.with.sensitivity.1"

"1",5,5,5,5,5,5,5,5,5,5,5,5

"2",3.66,3.66,3.66,3.66,3.66,3.66,3.66,3.66,3.66,3.66,3.66,3.66

"3",5,5,5,5,3.66,5,2.33,5,5,5,2.33,5

"4",5,5,5,5,5,5,5,5,5,5,5,5

"5",3.66,3.66,5,5,3.66,3.66,5,5,3.66,3.66,5,3.66

"6",5,3.66,5,3.66,3.66,3.66,3.66,3.66,5,3.66,3.66,3.66

"7",5,5,5,3.66,3.66,3.66,5,5,5,3.66,3.66,3.66

"8",5,5,5,5,5,5,5,5,5,5,5,5

"9",5,5,5,5,5,5,5,5,5,5,5,5

"10",3.66,5,5,3.66,5,3.66,3.66,5,3.66,3.66,2.33,3.66

"11",5,5,5,2.33,3.66,3.66,3.66,5,3.66,3.66,2.33,3.66

"12",5,5,5,3.66,5,3.66,5,5,2.33,3.66,2.33,5

"13",5,5,5,5,5,2.33,3.66,5,5,3.66,5,3.66

"14",5,5,5,5,5,5,5,5,5,5,5,5

"15",5,5,5,5,5,3.66,3.66,5,5,3.66,2.33,5

"16",3.66,3.66,3.66,3.66,5,5,3.66,5,5,3.66,5,5

"17",5,3.66,5,3.66,3.66,5,5,3.66,3.66,5,3.66,5

"18",5,5,5,3.66,5,3.66,3.66,5,5,3.66,3.66,5

"19",5,3.66,5,2.33,5,3.66,3.66,3.66,3.66,3.66,5,5

"20",5,5,5,5,3.66,3.66,3.66,5,5,5,5,5

"21",5,5,5,5,5,5,5,5,5,5,5,5

"22",5,3.66,5,2.33,3.66,5,5,5,2.33,2.33,5,5

"23",3.66,2.33,5,3.66,2.33,2.33,2.33,5,2.33,5,3.66,2.33

"24",3.66,5,5,3.66,3.66,2.33,3.66,3.66,3.66,3.66,3.66,3.66

"25",3.66,5,5,2.33,3.66,2.33,5,5,5,5,1,2.33

"26",5,5,5,3.66,5,3.66,5,3.66,3.66,5,5,5

"27",2.33,3.66,3.66,3.66,3.66,2.33,3.66,5,3.66,3.66,5,3.66

"28",5,5,3.66,2.33,3.66,3.66,3.66,5,5,5,3.66,3.66

"29",5,5,5,5,5,5,5,5,5,5,5,5

"30",5,5,5,5,5,5,3.66,5,5,3.66,3.66,5

"31",3.66,3.66,5,2.33,3.66,3.66,2.33,5,3.66,2.33,3.66,3.66

"32",5,5,5,3.66,3.66,5,5,5,5,5,5,5

"33",5,5,5,5,5,5,5,5,5,5,5,5

"34",3.66,5,5,3.66,3.66,3.66,5,5,5,5,3.66,5

"35",5,5,5,5,5,5,5,5,5,5,5,5

"36",3.66,3.66,3.66,3.66,3.66,3.66,3.66,3.66,3.66,3.66,3.66,3.66

"37",3.66,3.66,5,3.66,5,2.33,3.66,5,5,5,2.33,3.66

"38",5,5,5,3.66,3.66,3.66,3.66,5,2.33,5,5,5

"39",3.66,5,5,5,5,3.66,5,5,5,3.66,5,5

"40",5,5,5,5,5,5,3.66,5,5,5,5,5

"41",5,5,5,5,5,5,5,5,5,5,3.66,5

"42",5,5,5,5,5,5,5,5,5,5,5,5

"43",3.66,5,5,3.66,2.33,2.33,3.66,5,3.66,3.66,5,5

"44",5,5,5,3.66,5,5,5,5,5,5,3.66,5

"45",5,3.66,3.66,5,3.66,3.66,5,3.66,5,3.66,3.66,5

"46",5,5,5,5,5,5,5,5,3.66,5,5,5

"47",3.66,5,5,3.66,5,3.66,2.33,5,5,5,3.66,3.66

"48",5,5,5,5,5,5,5,5,5,5,5,5

"49",3.66,5,5,3.66,5,3.66,3.66,3.66,3.66,3.66,3.66,3.66

"50",5,5,5,5,5,5,5,5,5,5,3.66,5

"51",5,5,5,5,5,5,5,5,5,5,5,5

"52",5,5,5,5,5,5,5,5,5,5,5,5

"53",5,5,5,5,5,5,3.66,5,5,3.66,5,5

"54",5,5,5,5,5,5,5,5,5,3.66,3.66,5

"55",5,5,5,2.33,3.66,3.66,3.66,5,2.33,5,5,5

"56",5,5,5,5,5,5,5,5,5,5,5,5

"57",5,5,3.66,3.66,3.66,2.33,5,3.66,5,3.66,3.66,3.66

"58",5,5,5,3.66,5,3.66,5,5,3.66,5,5,5

"59",3.66,5,5,5,3.66,2.33,2.33,5,5,5,5,3.66

"60",5,3.66,5,3.66,3.66,3.66,2.33,5,5,5,2.33,3.66

"61",3.66,3.66,3.66,3.66,3.66,2.33,5,5,3.66,5,2.33,3.66

"62",3.66,3.66,3.66,2.33,2.33,3.66,3.66,2.33,2.33,5,3.66,3.66

"63",5,5,5,3.66,3.66,2.33,2.33,5,2.33,5,3.66,5

"64",5,5,5,2.33,3.66,5,5,5,2.33,5,5,5

"65",5,5,5,5,5,5,5,5,5,5,5,5

"66",3.66,5,5,3.66,3.66,3.66,5,5,3.66,3.66,3.66,5

"67",5,5,3.66,5,5,3.66,3.66,5,3.66,2.33,3.66,5

"68",5,5,5,5,5,5,5,5,5,5,5,5

"69",5,5,5,5,3.66,5,5,5,3.66,3.66,5,5

"70",3.66,5,5,3.66,3.66,3.66,5,5,5,5,5,5

"71",3.66,5,5,3.66,3.66,2.33,3.66,5,3.66,3.66,3.66,3.66

"72",3.66,5,5,5,3.66,3.66,2.33,5,3.66,3.66,2.33,5

"73",5,5,5,5,5,5,5,5,5,5,5,5

"74",5,5,5,3.66,5,3.66,3.66,5,5,3.66,3.66,5

"75",5,3.66,3.66,2.33,5,2.33,3.66,5,3.66,3.66,3.66,5

"76",3.66,3.66,5,2.33,3.66,3.66,3.66,3.66,3.66,3.66,3.66,5

"77",5,5,5,3.66,3.66,5,5,5,3.66,5,5,5

"78",5,5,5,5,5,5,5,5,5,5,5,5

"79",5,5,5,3.66,3.66,3.66,3.66,5,3.66,5,5,5

"80",5,5,5,5,5,5,3.66,5,3.66,5,5,5

"81",2.33,3.66,3.66,3.66,3.66,2.33,3.66,3.66,5,3.66,3.66,3.66

"82",3.66,3.66,3.66,3.66,3.66,3.66,5,5,5,3.66,3.66,3.66

"83",5,5,5,5,5,3.66,5,5,3.66,3.66,5,5

"84",5,5,5,2.33,5,5,5,5,5,5,5,5

"85",5,5,5,5,5,5,5,5,5,5,5,5

"86",5,5,5,5,3.66,2.33,3.66,5,3.66,3.66,2.33,5

"87",5,5,3.66,3.66,3.66,3.66,3.66,5,3.66,5,3.66,3.66

"88",5,5,5,5,3.66,3.66,3.66,5,3.66,5,5,5

"89",5,5,5,5,5,5,5,3.66,5,3.66,3.66,3.66

"90",5,5,5,3.66,3.66,2.33,2.33,2.33,2.33,3.66,5,3.66

"91",3.66,5,3.66,3.66,3.66,5,5,3.66,3.66,5,3.66,3.66

"92",5,5,5,5,5,5,5,5,5,5,5,5

"93",5,5,5,3.66,5,3.66,5,2.33,5,3.66,5,5

"94",5,5,5,5,3.66,3.66,3.66,5,3.66,3.66,5,5

"95",5,3.66,3.66,2.33,3.66,3.66,3.66,5,2.33,3.66,2.33,3.66

"96",5,5,5,3.66,3.66,3.66,3.66,5,3.66,3.66,5,5

"97",3.66,3.66,5,2.33,5,2.33,2.33,5,2.33,3.66,2.33,5

"98",5,3.66,5,5,5,5,5,5,5,5,5,5

"99",5,5,5,3.66,3.66,5,5,3.66,3.66,3.66,5,5

"100",3.66,5,5,5,5,3.66,3.66,3.66,2.33,3.66,5,3.66

"101",5,5,5,5,5,5,5,5,5,3.66,3.66,5

"102",5,5,5,5,5,3.66,5,5,5,5,5,5

"103",3.66,3.66,5,3.66,5,3.66,3.66,5,5,3.66,5,5

"104",5,3.66,3.66,3.66,5,2.33,5,3.66,3.66,3.66,5,3.66

"105",5,5,5,5,5,5,5,5,5,3.66,5,3.66

"106",5,5,5,5,5,5,5,5,5,5,5,5

"107",5,5,5,3.66,3.66,3.66,5,5,5,3.66,2.33,5

"108",3.66,5,5,5,5,3.66,3.66,5,3.66,5,3.66,3.66

"109",5,5,5,2.33,5,2.33,2.33,5,3.66,5,5,5

"110",3.66,3.66,3.66,2.33,3.66,3.66,3.66,3.66,3.66,3.66,3.66,3.66

"111",3.66,3.66,5,3.66,5,5,3.66,5,3.66,5,3.66,5

"112",5,5,5,3.66,5,5,5,5,5,5,5,5

"113",2.33,3.66,3.66,2.33,3.66,2.33,3.66,5,3.66,2.33,3.66,3.66

"114",5,5,5,5,5,5,5,3.66,5,3.66,3.66,5

"115",5,3.66,5,3.66,3.66,3.66,3.66,5,5,3.66,5,5

"116",5,5,5,5,5,2.33,3.66,5,5,5,2.33,5

"117",5,5,5,5,5,5,3.66,3.66,2.33,5,2.33,5

"118",3.66,3.66,3.66,3.66,3.66,3.66,3.66,3.66,3.66,5,5,5

"119",5,5,5,5,5,3.66,3.66,5,3.66,3.66,3.66,5

"120",5,5,5,5,5,5,5,5,5,5,5,5

"121",3.66,5,5,5,5,5,5,5,5,5,5,5

## Fig 4: Difference between desired and perceived physician empathy (diff.PE) as a function of perceived physician empathy (pPE). X-Y scatterplot; horizontal lines denote equality between desired and perceived physician empathy plus or minus 1 standard deviation. Red curve is a cubic spline fit.

Raw data from which the visualisation was constructed:

"Case","diff.PE","perceived.PE"

"1",-1.66666666666667,3.33333333333333

"2",1.33333333333333,5

"3",0.305555555555555,4.75

"4",0,5

"5",-0.888888888888888,3.33333333333333

"6",0.416666666666667,4.41666666666667

"7",0.083333333333333,4.41666666666667

"8",-0.083333333333333,4.91666666666667

"9",-1,4

"10",1,5

"11",1.05555555555556,4.5

"12",-0.0555555555555545,4.16666666666667

"13",-0.527777777777778,3.91666666666667

"14",0,5

"15",0.305555555555555,4.75

"16",0.249999999999999,4.58333333333333

"17",0.333333333333333,4.66666666666667

"18",-0.111111111111112,4.33333333333333

"19",-0.0277777777777777,4.08333333333333

"20",-0.083333333333333,4.58333333333333

"21",0,5

"22",0.138888888888889,4.25

"23",-0.416666666666667,2.91666666666667

"24",0.305555555555555,4.08333333333333

"25",-0.444444444444445,3.33333333333333

"26",0.111111111111112,4.66666666666667

"27",0,3.66666666666667

"28",-0.444444444444444,3.66666666666667

"29",1.77777777777778,5

"30",-0.416666666666666,4.25

"31",0.444444444444444,4

"32",0.0555555555555545,4.83333333333333

"33",-0.25,4.75

"34",-0.194444444444445,4.25

"35",-1.41666666666667,3.58333333333333

"36",1.33333333333333,5

"37",-1.33333333333333,2.66666666666667

"38",-0.166666666666667,4.16666666666667

"39",0.333333333333334,5

"40",-0.222222222222222,4.66666666666667

"41",0.111111111111111,5

"42",-1.83333333333333,3.16666666666667

"43",-1.91666666666667,2.08333333333333

"44",-0.861111111111112,3.91666666666667

"45",-1.72222222222222,2.5

"46",-0.222222222222222,4.66666666666667

"47",-0.555555555555555,3.66666666666667

"48",-1.83333333333333,3.16666666666667

"49",0.75,4.75

"50",-0.138888888888889,4.75

"51",-0.083333333333333,4.91666666666667

"52",-3.16666666666667,1.83333333333333

"53",-0.777777777777779,4

"54",-3.02777777777778,1.75

"55",-1.72222222222222,2.5

"56",0,5

"57",0.166666666666667,4.16666666666667

"58",0.0833333333333339,4.75

"59",-0.638888888888888,3.58333333333333

"60",-1.41666666666667,2.58333333333333

"61",-0.861111111111112,2.91666666666667

"62",1.66666666666667,5

"63",0.916666666666667,4.91666666666667

"64",-0.0277777777777777,4.41666666666667

"65",0,5

"66",0.277777777777779,4.5

"67",-0.972222222222221,3.25

"68",-0.583333333333333,4.41666666666667

"69",-0.583333333333333,4.08333333333333

"70",-1.80555555555556,2.75

"71",-0.388888888888889,3.5

"72",0.583333333333333,4.58333333333333

"73",0,5

"74",0.305555555555555,4.75

"75",-0.222222222222222,3.66666666666667

"76",0.0555555555555554,3.83333333333333

"77",0.333333333333334,5

"78",-3.66666666666667,1.33333333333333

"79",-1.61111111111111,2.83333333333333

"80",0.222222222222221,5

"81",-1.30555555555556,2.25

"82",-1.25,2.75

"83",-0.499999999999999,4.16666666666667

"84",-0.527777777777779,4.25

"85",-2.91666666666667,2.08333333333333

"86",-1.27777777777778,2.83333333333333

"87",-1.11111111111111,3

"88",-0.638888888888889,3.91666666666667

"89",-0.388888888888888,4.16666666666667

"90",0.833333333333333,4.5

"91",0.555555555555556,4.66666666666667

"92",0,5

"93",-0.944444444444445,3.5

"94",-1.19444444444444,3.25

"95",0.194444444444444,3.75

"96",-0.250000000000001,4.08333333333333

"97",-0.638888888888889,2.91666666666667

"98",-0.138888888888889,4.75

"99",0.472222222222222,4.91666666666667

"100",-1.44444444444444,2.66666666666667

"101",-0.944444444444445,3.83333333333333

"102",-2.63888888888889,2.25

"103",-2.08333333333333,2.25

"104",-1.41666666666667,2.58333333333333

"105",-0.861111111111112,3.91666666666667

"106",0,5

"107",0.666666666666666,5

"108",0.666666666666666,5

"109",-0.972222222222221,3.25

"110",-0.805555555555556,2.75

"111",0.416666666666666,4.75

"112",-0.138888888888889,4.75

"113",0.5,3.83333333333333

"114",-0.083333333333333,4.58333333333333

"115",0.666666666666666,5

"116",-2.27777777777778,2.16666666666667

"117",0.416666666666666,4.75

"118",0.166666666666667,4.16666666666667

"119",-1.19444444444444,3.25

"120",-3.16666666666667,1.83333333333333

"121",-2.55555555555556,2.33333333333333

## Fig 5: Patient satisfaction with the physician as a function of the difference between desired and perceived physician empathy (diff.PE); X-Y scatterplot. The line represents a cubic smoothing spline fit.

Raw data from which the visualisation was constructed:

"Case","Patient satisfaction","diff.PE"

"1",3,-1.66666666666667

"2",4,1.33333333333333

"3",4,0.305555555555555

"4",3.5,0

"5",3,-0.888888888888888

"6",3,0.416666666666667

"7",3.5,0.083333333333333

"8",4,-0.083333333333333

"9",4,-1

"10",4,1

"11",3.5,1.05555555555556

"12",3,-0.0555555555555545

"13",3,-0.527777777777778

"14",4,0

"15",3.5,0.305555555555555

"16",3.5,0.249999999999999

"17",4,0.333333333333333

"18",4,-0.111111111111112

"19",3,-0.0277777777777777

"20",4,-0.083333333333333

"21",4,0

"22",3.5,0.138888888888889

"23",2.5,-0.416666666666667

"24",3.5,0.305555555555555

"25",3,-0.444444444444445

"26",3.5,0.111111111111112

"27",3.5,0

"28",3,-0.444444444444444

"29",4,1.77777777777778

"30",3,-0.416666666666666

"31",3,0.444444444444444

"32",4,0.0555555555555545

"33",4,-0.25

"34",4,-0.194444444444445

"35",3,-1.41666666666667

"36",4,1.33333333333333

"37",3,-1.33333333333333

"38",3,-0.166666666666667

"39",4,0.333333333333334

"40",4,-0.222222222222222

"41",4,0.111111111111111

"42",3,-1.83333333333333

"43",3,-1.91666666666667

"44",3,-0.861111111111112

"45",2.5,-1.72222222222222

"46",4,-0.222222222222222

"47",3,-0.555555555555555

"48",4,-1.83333333333333

"49",3.5,0.75

"50",3.5,-0.138888888888889

"51",4,-0.083333333333333

"52",2,-3.16666666666667

"53",3,-0.777777777777779

"54",2,-3.02777777777778

"55",3.5,-1.72222222222222

"56",4,0

"57",3.5,0.166666666666667

"58",4,0.0833333333333339

"59",2,-0.638888888888888

"60",3,-1.41666666666667

"61",3,-0.861111111111112

"62",4,1.66666666666667

"63",4,0.916666666666667

"64",4,-0.0277777777777777

"65",4,0

"66",4,0.277777777777779

"67",2.5,-0.972222222222221

"68",3,-0.583333333333333

"69",4,-0.583333333333333

"70",2,-1.80555555555556

"71",3,-0.388888888888889

"72",3,0.583333333333333

"73",4,0

"74",3.5,0.305555555555555

"75",3.5,-0.222222222222222

"76",3.5,0.0555555555555554

"77",4,0.333333333333334

"78",1,-3.66666666666667

"79",2,-1.61111111111111

"80",4,0.222222222222221

"81",3.5,-1.30555555555556

"82",2.5,-1.25

"83",4,-0.499999999999999

"84",4,-0.527777777777779

"85",3,-2.91666666666667

"86",3,-1.27777777777778

"87",3,-1.11111111111111

"88",4,-0.638888888888889

"89",3.5,-0.388888888888888

"90",4,0.833333333333333

"91",3.5,0.555555555555556

"92",4,0

"93",3,-0.944444444444445

"94",3.5,-1.19444444444444

"95",3.5,0.194444444444444

"96",4,-0.250000000000001

"97",3,-0.638888888888889

"98",4,-0.138888888888889

"99",3.5,0.472222222222222

"100",2.5,-1.44444444444444

"101",4,-0.944444444444445

"102",2.5,-2.63888888888889

"103",2.5,-2.08333333333333

"104",2.5,-1.41666666666667

"105",3.5,-0.861111111111112

"106",3.5,0

"107",4,0.666666666666666

"108",4,0.666666666666666

"109",3,-0.972222222222221

"110",3,-0.805555555555556

"111",3.5,0.416666666666666

"112",4,-0.138888888888889

"113",3.5,0.5

"114",3.5,-0.083333333333333

"115",4,0.666666666666666

"116",2.5,-2.27777777777778

"117",4,0.416666666666666

"118",4,0.166666666666667

"119",3.5,-1.19444444444444

"120",1,-3.16666666666667

"121",2,-2.55555555555556

## Fig 6: Association between trust in the physician (Trust) and the difference between desired and perceived physician empathy (diff.PE); X-Y scatterplot. The line represents a cubic smoothing spline fit.

Raw data from which the visualisation was constructed:

"Case"," Trust ","diff.PE"

"1",4,-1.66666666666667

"2",5,1.33333333333333

"3",4.66666666666667,0.305555555555555

"4",5,0

"5",3.66666666666667,-0.888888888888888

"6",4,0.416666666666667

"7",3.66666666666667,0.083333333333333

"8",4.66666666666667,-0.083333333333333

"9",5,-1

"10",4.66666666666667,1

"11",4.33333333333333,1.05555555555556

"12",5,-0.0555555555555545

"13",4,-0.527777777777778

"14",5,0

"15",4.66666666666667,0.305555555555555

"16",4.66666666666667,0.249999999999999

"17",4,0.333333333333333

"18",5,-0.111111111111112

"19",4,-0.0277777777777777

"20",4.33333333333333,-0.083333333333333

"21",5,0

"22",4.33333333333333,0.138888888888889

"23",3,-0.416666666666667

"24",4.33333333333333,0.305555555555555

"25",4.33333333333333,-0.444444444444445

"26",3.66666666666667,0.111111111111112

"27",4.33333333333333,0

"28",4.33333333333333,-0.444444444444444

"29",5,1.77777777777778

"30",4.66666666666667,-0.416666666666666

"31",4.66666666666667,0.444444444444444

"32",4.33333333333333,0.0555555555555545

"33",5,-0.25

"34",5,-0.194444444444445

"35",5,-1.41666666666667

"36",5,1.33333333333333

"37",3.33333333333333,-1.33333333333333

"38",4.33333333333333,-0.166666666666667

"39",5,0.333333333333334

"40",5,-0.222222222222222

"41",5,0.111111111111111

"42",4,-1.83333333333333

"43",4,-1.91666666666667

"44",4.33333333333333,-0.861111111111112

"45",4,-1.72222222222222

"46",5,-0.222222222222222

"47",4,-0.555555555555555

"48",5,-1.83333333333333

"49",4.66666666666667,0.75

"50",4,-0.138888888888889

"51",4.66666666666667,-0.083333333333333

"52",2.33333333333333,-3.16666666666667

"53",4.66666666666667,-0.777777777777779

"54",3.66666666666667,-3.02777777777778

"55",4,-1.72222222222222

"56",4.33333333333333,0

"57",4.66666666666667,0.166666666666667

"58",4.66666666666667,0.0833333333333339

"59",3,-0.638888888888888

"60",4,-1.41666666666667

"61",4,-0.861111111111112

"62",5,1.66666666666667

"63",5,0.916666666666667

"64",5,-0.0277777777777777

"65",5,0

"66",4.66666666666667,0.277777777777779

"67",3.66666666666667,-0.972222222222221

"68",4,-0.583333333333333

"69",5,-0.583333333333333

"70",2.66666666666667,-1.80555555555556

"71",3,-0.388888888888889

"72",3.66666666666667,0.583333333333333

"73",4.66666666666667,0

"74",4.66666666666667,0.305555555555555

"75",2.66666666666667,-0.222222222222222

"76",4.66666666666667,0.0555555555555554

"77",4.66666666666667,0.333333333333334

"78",2.33333333333333,-3.66666666666667

"79",3.66666666666667,-1.61111111111111

"80",4.66666666666667,0.222222222222221

"81",4,-1.30555555555556

"82",3.33333333333333,-1.25

"83",4,-0.499999999999999

"84",5,-0.527777777777779

"85",3,-2.91666666666667

"86",4.33333333333333,-1.27777777777778

"87",4,-1.11111111111111

"88",4.33333333333333,-0.638888888888889

"89",5,-0.388888888888888

"90",4,0.833333333333333

"91",5,0.555555555555556

"92",4.66666666666667,0

"93",4.66666666666667,-0.944444444444445

"94",4.66666666666667,-1.19444444444444

"95",4.33333333333333,0.194444444444444

"96",4.33333333333333,-0.250000000000001

"97",3.66666666666667,-0.638888888888889

"98",4.66666666666667,-0.138888888888889

"99",4,0.472222222222222

"100",4,-1.44444444444444

"101",3.66666666666667,-0.944444444444445

"102",2.66666666666667,-2.63888888888889

"103",3,-2.08333333333333

"104",4,-1.41666666666667

"105",4,-0.861111111111112

"106",5,0

"107",4.66666666666667,0.666666666666666

"108",4.66666666666667,0.666666666666666

"109",4.33333333333333,-0.972222222222221

"110",3,-0.805555555555556

"111",5,0.416666666666666

"112",4.33333333333333,-0.138888888888889

"113",4.66666666666667,0.5

"114",3.33333333333333,-0.083333333333333

"115",4.66666666666667,0.666666666666666

"116",4.33333333333333,-2.27777777777778

"117",5,0.416666666666666

"118",4,0.166666666666667

"119",4.33333333333333,-1.19444444444444

"120",2.66666666666667,-3.16666666666667

"121",2.66666666666667,-2.55555555555556

## Fig 7: Association between trust in the physician (Trust) and patient satisfaction; X-Y scatterplot. The line represents a cubic smoothing spline fit.

Raw data from which the visualisation was constructed:

"Case","Satisfaction ","Trust"

"1",3,4

"2",4,5

"3",4,4.66666666666667

"4",3.5,5

"5",3,3.66666666666667

"6",3,4

"7",3.5,3.66666666666667

"8",4,4.66666666666667

"9",4,5

"10",4,4.66666666666667

"11",3.5,4.33333333333333

"12",3,5

"13",3,4

"14",4,5

"15",3.5,4.66666666666667

"16",3.5,4.66666666666667

"17",4,4

"18",4,5

"19",3,4

"20",4,4.33333333333333

"21",4,5

"22",3.5,4.33333333333333

"23",2.5,3

"24",3.5,4.33333333333333

"25",3,4.33333333333333

"26",3.5,3.66666666666667

"27",3.5,4.33333333333333

"28",3,4.33333333333333

"29",4,5

"30",3,4.66666666666667

"31",3,4.66666666666667

"32",4,4.33333333333333

"33",4,5

"34",4,5

"35",3,5

"36",4,5

"37",3,3.33333333333333

"38",3,4.33333333333333

"39",4,5

"40",4,5

"41",4,5

"42",3,4

"43",3,4

"44",3,4.33333333333333

"45",2.5,4

"46",4,5

"47",3,4

"48",4,5

"49",3.5,4.66666666666667

"50",3.5,4

"51",4,4.66666666666667

"52",2,2.33333333333333

"53",3,4.66666666666667

"54",2,3.66666666666667

"55",3.5,4

"56",4,4.33333333333333

"57",3.5,4.66666666666667

"58",4,4.66666666666667

"59",2,3

"60",3,4

"61",3,4

"62",4,5

"63",4,5

"64",4,5

"65",4,5

"66",4,4.66666666666667

"67",2.5,3.66666666666667

"68",3,4

"69",4,5

"70",2,2.66666666666667

"71",3,3

"72",3,3.66666666666667

"73",4,4.66666666666667

"74",3.5,4.66666666666667

"75",3.5,2.66666666666667

"76",3.5,4.66666666666667

"77",4,4.66666666666667

"78",1,2.33333333333333

"79",2,3.66666666666667

"80",4,4.66666666666667

"81",3.5,4

"82",2.5,3.33333333333333

"83",4,4

"84",4,5

"85",3,3

"86",3,4.33333333333333

"87",3,4

"88",4,4.33333333333333

"89",3.5,5

"90",4,4

"91",3.5,5

"92",4,4.66666666666667

"93",3,4.66666666666667

"94",3.5,4.66666666666667

"95",3.5,4.33333333333333

"96",4,4.33333333333333

"97",3,3.66666666666667

"98",4,4.66666666666667

"99",3.5,4

"100",2.5,4

"101",4,3.66666666666667

"102",2.5,2.66666666666667

"103",2.5,3

"104",2.5,4

"105",3.5,4

"106",3.5,5

"107",4,4.66666666666667

"108",4,4.66666666666667

"109",3,4.33333333333333

"110",3,3

"111",3.5,5

"112",4,4.33333333333333

"113",3.5,4.66666666666667

"114",3.5,3.33333333333333

"115",4,4.66666666666667

"116",2.5,4.33333333333333

"117",4,5

"118",4,4

"119",3.5,4.33333333333333

"120",1,2.66666666666667

"121",2,2.66666666666667

## Fig 8: Tukey’s box plots with a notch depicting the subjective burden of various aspects of the disease and their ratings

Raw data from which the visualisation was constructed:

"Case","Overall..because.of.your.intestinal.disease.or.symptoms","Your.worries.about.your.disease","Embarrassment.associated.with.intestinal.symptoms","Procedures.and.treatments.for.your.disease","Examinations.performed.because.of..your.disease","Impairments.or.limitations.in.everyday.life","Physical.problems.and.symptoms"

"1",3,3,4,3,2,4,4

"2",2,2,2,2,2,2,2

"3",4,1,2,4,4,3,3

"4",3,3,3,3,3,3,3

"5",3,2,4,3,2,4,4

"6",4,3,3,2,2,4,4

"7",3,3,4,4,3,4,4

"8",3,4,2,4,3,3,2

"9",4,4,3,3,2,4,4

"10",1,2,1,1,1,1,1

"11",3,3,3,1,1,3,3

"12",3,4,1,3,1,2,3

"13",3,4,3,3,3,4,4

"14",1,4,1,4,1,3,4

"15",1,2,2,2,2,1,1

"16",2,1,1,2,1,2,1

"17",3,3,3,4,3,3,3

"18",4,4,4,4,4,4,4

"19",2,3,2,3,3,3,3

"20",3,3,4,3,2,3,2

"21",4,4,4,3,3,4,4

"22",3,3,2,2,2,2,2

"23",2,2,2,2,2,2,2

"24",3,4,3,3,2,2,3

"25",2,3,2,1,1,2,2

"26",3,4,4,4,4,3,3

"27",2,2,2,3,3,2,3

"28",2,3,2,1,2,3,2

"29",1,1,1,1,1,1,1

"30",2,2,2,3,3,1,2

"31",3,3,2,3,2,3,3

"32",3,4,3,4,1,2,3

"33",3,3,3,4,3,3,3

"34",3,3,2,3,4,2,3

"35",4,3,3,3,2,3,3

"36",1,2,2,3,2,1,1

"37",3,1,1,2,2,4,4

"38",3,3,3,4,3,4,3

"39",4,4,4,3,2,4,4

"40",3,3,3,2,3,3,3

"41",4,4,3,4,4,3,4

"42",4,3,2,3,2,4,4

"43",4,3,3,4,4,4,4

"44",3,3,2,3,3,3,4

"45",3,4,2,4,3,3,4

"46",3,3,4,4,3,3,3

"47",4,4,4,3,2,4,4

"48",4,4,4,3,3,4,4

"49",3,3,1,2,2,4,4

"50",3,3,2,4,3,4,4

"51",3,3,4,2,2,3,3

"52",3,4,3,4,4,3,4

"53",4,3,2,3,2,4,3

"54",3,3,3,3,4,4,4

"55",3,3,3,1,1,2,3

"56",3,3,4,3,4,3,3

"57",3,4,3,2,2,4,3

"58",2,3,2,3,2,2,2

"59",3,4,4,3,3,4,4

"60",4,3,4,3,3,3,3

"61",3,3,2,3,3,4,3

"62",4,4,3,4,4,4,4

"63",2,2,4,1,1,2,3

"64",3,2,1,4,4,3,2

"65",3,3,2,4,3,3,3

"66",3,3,2,2,3,2,3

"67",4,2,4,3,4,4,4

"68",3,3,4,3,4,3,3

"69",3,3,2,3,3,4,4

"70",3,3,3,2,2,3,3

"71",2,2,2,4,3,3,4

"72",4,4,1,4,3,4,4

"73",4,4,4,2,2,4,3

"74",3,3,2,2,2,3,3

"75",4,3,4,3,3,4,3

"76",2,1,2,2,1,2,2

"77",3,2,2,2,3,3,2

"78",4,4,2,4,4,3,4

"79",3,4,3,2,2,3,3

"80",4,4,1,3,2,4,4

"81",4,4,4,4,4,3,3

"82",3,3,2,3,2,3,3

"83",3,2,4,4,4,3,3

"84",4,4,2,3,2,4,4

"85",3,3,4,4,3,4,4

"86",4,4,3,3,3,3,3

"87",3,3,4,4,3,4,4

"88",3,4,4,4,3,3,3

"89",3,4,2,2,2,3,3

"90",3,4,4,4,4,2,2

"91",3,3,2,2,2,4,3

"92",3,4,1,2,1,2,3

"93",3,4,2,2,2,4,3

"94",3,4,3,4,4,3,3

"95",1,1,3,2,2,1,1

"96",3,3,4,4,2,3,3

"97",3,3,2,3,2,3,4

"98",4,4,3,4,4,4,4

"99",3,4,2,3,3,2,2

"100",4,2,4,4,3,4,4

"101",4,3,2,4,3,4,4

"102",3,3,2,4,4,3,3

"103",4,3,4,3,3,3,2

"104",2,4,2,4,3,2,3

"105",3,3,2,2,2,3,3

"106",4,4,2,4,4,4,4

"107",3,2,2,2,2,3,4

"108",3,3,4,4,2,3,3

"109",4,4,4,3,4,3,3

"110",4,4,2,4,4,3,4

"111",3,4,3,3,3,3,4

"112",1,1,1,1,1,1,2

"113",3,2,2,2,2,3,3

"114",3,2,2,3,2,3,3

"115",2,2,2,2,2,1,2

"116",3,4,3,4,3,4,4

"117",2,1,3,2,1,3,3

"118",2,3,4,4,3,2,3

"119",3,4,1,4,4,3,3

"120",3,3,4,2,3,3,4

"121",3,3,3,4,4,3,3

## Fig 9: Tukey’s box plots with a notch depicting patient resources and their ratings

Raw data from which the visualisation was constructed:

"Case","Family and friends","A good relationship with my physician","Plans and goals I set myself","Distraction through sport/activities","Relaxation","My belief/religion/spirituality","The search for information about my disease"

"1",5,5,4,4,5,3,3

"2",4,4,4,5,4,4,3

"3",5,5,5,5,3,1,1

"4",5,5,5,5,5,5,5

"5",4,4,3,3,3,2,2

"6",5,5,4,1,2,1,2

"7",4,4,4,4,4,2,3

"8",5,5,4,3,3,5,2

"9",2,2,4,1,1,4,3

"10",5,4,3,5,3,2,1

"11",4,4,5,5,5,1,2

"12",5,3,2,3,2,1,5

"13",5,4,5,5,4,1,2

"14",5,5,5,1,3,1,1

"15",5,4,3,3,5,2,2

"16",5,4,3,2,4,1,1

"17",5,5,4,4,5,1,3

"18",5,5,5,5,5,5,5

"19",5,4,4,4,4,4,3

"20",5,5,3,5,4,1,2

"21",5,5,3,1,1,1,1

"22",4,3,2,5,5,1,3

"23",3,3,4,4,4,1,2

"24",5,4,2,4,5,2,3

"25",2,4,5,4,4,2,4

"26",5,3,4,5,3,1,1

"27",4,3,5,5,4,2,1

"28",5,4,5,5,5,3,4

"29",5,NA,5,NA,5,5,5

"30",4,5,3,2,4,1,2

"31",4,3,2,5,4,2,1

"32",5,4,4,3,3,3,2

"33",5,5,5,5,5,1,5

"34",5,5,3,3,3,2,2

"35",5,4,3,4,5,3,3

"36",5,4,1,4,4,1,1

"37",5,3,3,2,3,1,2

"38",5,4,4,4,4,1,4

"39",5,5,3,2,3,1,4

"40",5,5,4,4,5,1,3

"41",5,5,5,3,4,3,3

"42",5,1,1,1,1,1,4

"43",3,4,3,2,5,2,5

"44",5,5,5,5,5,4,5

"45",5,4,3,3,4,1,4

"46",4,3,4,5,3,2,3

"47",4,3,5,3,3,1,3

"48",4,4,4,4,5,4,5

"49",5,4,4,3,2,1,2

"50",5,5,5,4,5,5,5

"51",5,5,4,4,5,1,4

"52",5,2,5,5,5,1,4

"53",1,3,2,4,3,2,4

"54",4,2,4,4,5,5,5

"55",5,4,1,5,4,1,5

"56",5,5,4,3,5,1,4

"57",5,5,4,5,3,1,3

"58",5,4,4,3,4,2,3

"59",5,3,5,3,5,1,3

"60",5,4,4,5,4,1,3

"61",4,3,4,3,2,1,3

"62",5,5,5,2,5,1,5

"63",3,4,5,4,5,5,4

"64",5,5,4,4,3,2,5

"65",4,5,5,5,5,1,4

"66",3,4,4,3,4,2,3

"67",5,5,3,4,3,1,4

"68",5,5,5,4,4,4,3

"69",5,5,5,5,5,3,3

"70",3,2,4,5,5,2,4

"71",5,3,2,2,5,1,4

"72",5,5,2,2,2,1,3

"73",5,5,4,4,5,3,5

"74",5,4,3,3,3,1,3

"75",5,3,2,3,3,1,4

"76",4,4,3,3,3,5,5

"77",5,5,3,3,4,3,4

"78",2,1,4,3,5,4,2

"79",4,3,4,4,3,2,4

"80",2,5,3,4,5,1,5

"81",3,3,4,4,5,1,3

"82",4,3,4,4,4,2,4

"83",3,3,4,4,4,1,1

"84",5,5,4,3,3,5,4

"85",2,5,5,3,5,2,3

"86",4,4,4,5,5,1,3

"87",5,4,2,2,3,1,3

"88",5,4,4,4,4,2,5

"89",5,4,3,3,5,3,3

"90",5,4,4,4,5,2,4

"91",4,4,3,3,4,1,2

"92",1,5,3,2,1,1,5

"93",3,5,2,3,2,5,5

"94",4,4,4,5,5,2,4

"95",5,3,2,3,4,5,4

"96",5,4,5,4,5,2,2

"97",4,2,3,5,4,1,3

"98",5,5,5,3,4,2,3

"99",5,5,4,5,4,5,5

"100",5,3,3,2,4,2,3

"101",4,3,3,2,4,2,3

"102",5,5,3,2,2,1,5

"103",3,1,1,3,1,1,1

"104",5,3,5,5,5,3,3

"105",3,3,4,4,4,3,4

"106",5,5,4,2,2,1,4

"107",5,5,5,3,5,1,2

"108",4,4,3,2,3,2,4

"109",4,3,3,2,2,1,5

"110",2,2,3,3,3,1,5

"111",5,5,4,5,5,1,5

"112",5,5,3,1,1,3,5

"113",5,4,4,4,4,5,2

"114",4,3,4,5,5,5,3

"115",5,5,5,5,5,2,2

"116",2,3,1,4,4,3,3

"117",5,5,4,5,3,1,2

"118",5,4,5,4,4,2,3

"119",2,3,4,4,4,2,4

"120",5,1,3,1,1,1,5

"121",5,3,4,4,5,1,2
